# Putting Hybrid Nanomaterials to Work for Biomedical Applications

**DOI:** 10.1002/anie.202319567

**Published:** 2024-03-15

**Authors:** Dong Zhang, Yidan Chen, Min Hao, Younan Xia

**Affiliations:** The Wallace H. Coulter Department of Biomedical Engineering, Georgia Institute of Technology and Emory University, Atlanta, GA 30332 (USA); School of Materials Science and Engineering, Georgia Institute of Technology, Atlanta, GA 30332 (USA); The Wallace H. Coulter Department of Biomedical Engineering, Georgia Institute of Technology and Emory University, Atlanta, GA 30332 (USA); The Wallace H. Coulter Department of Biomedical Engineering, Georgia Institute of Technology and Emory University, Atlanta, GA 30332 (USA); School of Chemistry and Biochemistry, Georgia Institute of Technology, Atlanta, GA 30332 (USA)

**Keywords:** nanomaterials, hybridization, nanomedicine, regenerative medicine, theranostics

## Abstract

Hybrid nanomaterials have found use in many biomedical applications. This article provides a comprehensive review of the principles, techniques, and recent advancements in the design and fabrication of hybrid nanomaterials for biomedicine. We begin with an introduction to the general concept of material hybridization, followed by a discussion of how this approach leads to materials with additional functionality and enhanced performance. We then highlight hybrid nanomaterials in the forms of nanostructures, nanocomposites, metal–organic frameworks, and biohybrids, including their fabrication methods. We also showcase the use of hybrid nanomaterials to advance biomedical engineering in the context of nanomedicine, regenerative medicine, diagnostics, theranostics, and biomanufacturing. Finally, we offer perspectives on challenges and opportunities.

## Graphical Abstract

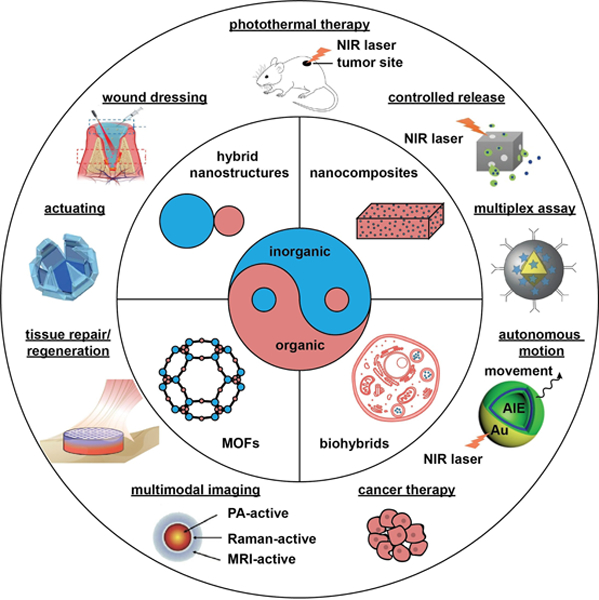

## 1. Introduction

Materials can be divided into two major categories–inorganic and organic–with their difference being the exclusion and inclusion of carbon, respectively.^[1]^ However, materials made of elementary carbon and compounds of carbon with, for example, nitrogen, oxygen, or silicon are traditionally classified as inorganic rather than organic. Examples include carbon materials (e.g., fullerenes, diamond, graphite, graphene, and carbon nanotubes),^[2]^ SiC, CO, CO_2_, as well as H_2_CO_3_ and salts thereof. All other types of carbon-containing substances are organic materials. Polymers are typically categorized as organic materials albeit inorganic polymers with backbones comprised of Si–Si bonds (polysilanes), Si–O (polysiloxanes), and P–N (polyphosphazenes) are also well known.^[3]^ Both inorganic and organic materials can be engineered to provide similar electronic, electrical, and optical (e.g., scattering, absorption, and light-emitting) properties, but they exhibit intrinsic distinctions. Organic materials, for example, are superior in biocompatibility and biodegradability, in addition to their unique capability to achieve selective binding (for the purpose of targeting)^[4]^ and self-replication.^[5]^ They are also known for being much lighter and softer than their inorganic counterparts, in addition to their much lower melting points and the stronger tendency of polymers to take an amorphous structure. Some applications such as those involving magnetism are still dominated by inorganic materials. As for biomedical applications, inorganic and organic materials tend to possess complementary capabilities, making it advantageous to integrate them into a hybrid system.^[6]^ In recent decades, the concept of hybridization has been productively extended from bulk to nanoscale to access the full spectrum of properties offered by diverse materials on different length scales.

**Figure 1.**
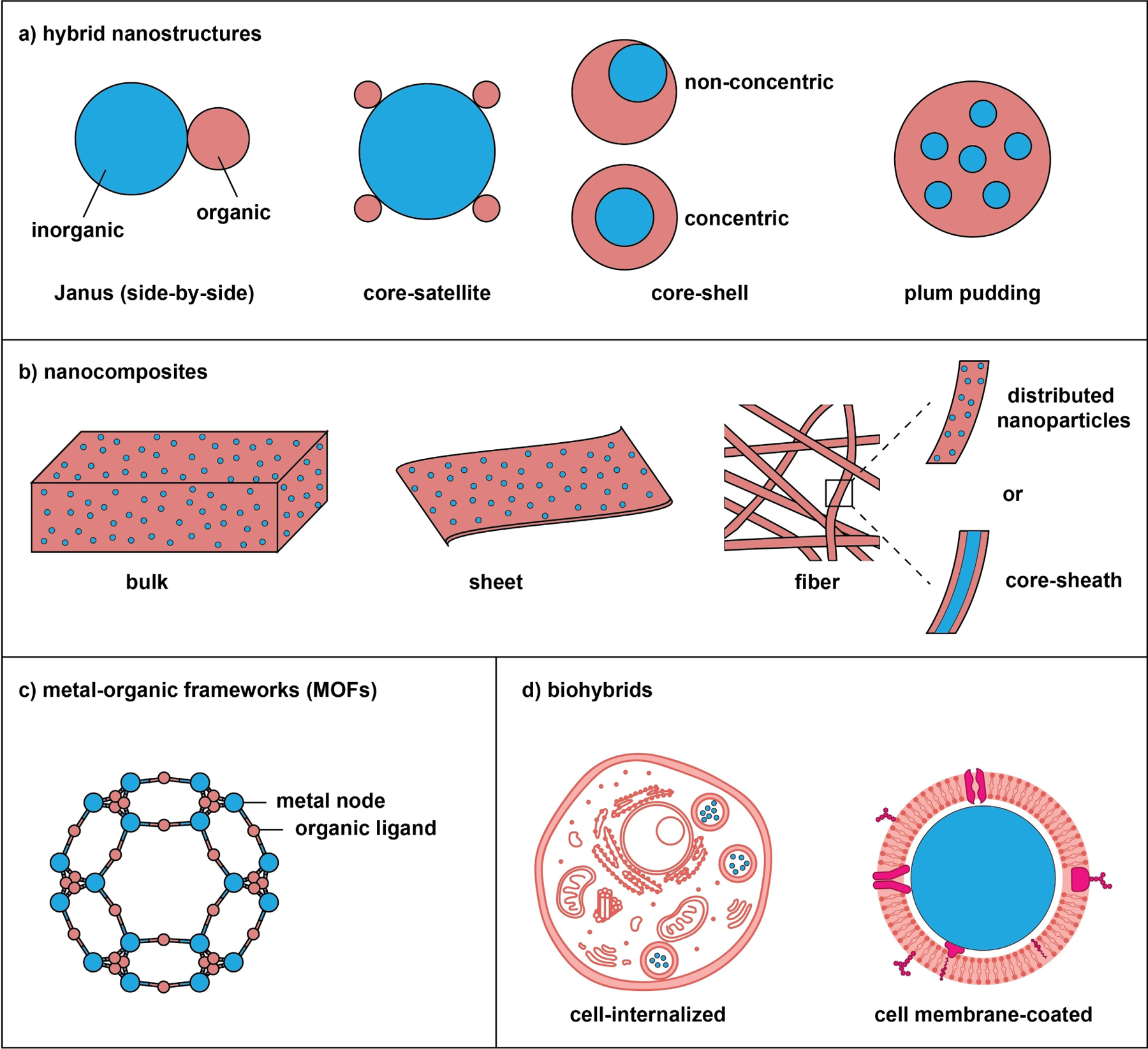
Schematics of four major classes of hybrid nanomaterials. For hybrid nanostructures, the inorganic and organic components can be arranged in various configurations whereas both nanocomposites and MOFs can be processed into different morphologies.

For simplicity and consistency, we define hybrid nanomaterials as structures with at least one of the two constituents (typically, the inorganic component) having one or more of its dimensions controlled in the range of 1–100 nm. This definition leads to two classes of hybrid nanomaterials (Figure 1): hybrid nanostructures (with both components on the nanoscale) and nanocomposites (normally, with the inorganic component on the nanoscale). Metal–organic frameworks (MOFs) represent a different class, which involves the use of inorganic and organic components in the ionic or molecular formats and the final structure has at least one dimension in the range of 1–100 nm. In recent years, another class (i.e., biohybrids) also emerged, in which inorganic nanostructures were loaded into live cells or encapsulated by the membranes derived from cells. In principle, the inorganic and organic components can be arranged in different configurations to expand the diversity and functionality.

Hybridization is a prerequisite in the preparation and/or utilization of essentially all types of inorganic nanomaterials due to their high specific surface energies.^[7]^ They cannot exist as stable suspensions in liquids unless their surface is passivated by a colloidal stabilizer in the form of ions, small molecules, or polymers.^[8]^ The biocompatibility, pharmacokinetics, and biodistribution of inorganic nanomaterials can also be optimized through surface modification with organic ligands^[9]^. Some inorganic nanomaterials are highly cytotoxic, while others, though bio-inert, can cause adverse impacts if accumulated in healthy tissues such as the spleen, liver, and kidneys.^[10]^ Conversely, the treatment efficacy of a nanomedicine will be compromised if it is quickly excreted from the body.^[11]^ Taken together, it is vital to modify the surface of inorganic nanomaterials with organic ligands, endowing them with in vivo safety while ensuring desirable interactions with the biological system. In this regard, hybridization holds the key to biomedical applications, including augmented performance, new capabilities, and multi-functionality.

The concept of hybridization is widely exploited by the biological world to achieve specific functionality.^[12]^ For example, many proteins or enzymes are not purely made of amino acids. They also contain inorganic components such as metal ions. A notable example is hemoglobin, the iron-containing protein present in red blood cells of almost all vertebrates that is responsible for the transportation of O_2_, CO_2_, and other species.^[13]^ It is the intricate interplay between the Fe^2+^/Fe^3+^ ion, porphyrin ring, histidine units, and the entire protein backbone that makes it possible to deliver O_2_ from the lung to the tissues while carrying back the CO_2_ at remarkable efficiency. As another example, bone is a composite material containing cells, proteins, collagens, and inorganic minerals, especially those involving Ca^2+^ and PO_4_^3−^. The mineral phase makes bone hard and strong while the collagen fibers provide flexibility so that the bone can resist breaking. At the tendon-to-bone enthesis, in particular, there exist gradations in composition, structure, and cell phenotype.^[14]^ Across a distance of ca. 100 μm, the enthesis contains four compositionally distinct but structurally continuous zones, including tendon, unmineralized fibrocartilage, mineralized fibrocartilage, and bone. Each zone is unique in terms of mineral content, cellular type, extracellular matrix (ECM), and collagen alignment. Such graded transitions play a vital role in eliminating stress concentrations that would otherwise arise from the mismatch in mechanical property between tendon (with a modulus of ca. 200 MPa) and bone (with a modulus of ca. 20 GPa). Once injured, it is difficult to regenerate the enthesis during healing, even with surgical intervention.^[15,16]^ A promising procedure involves the use of a graded nanocomposite to promote healing and functional recovery by serving as a biomimetic scaffold to duplicate various structural aspects of the native enthesis.^[17]^

There are countless good examples of hybrid nanomaterials. The inorganic components may include metal ions/atoms/clusters, inorganic nanostructures, and carbon nanomaterials. In the broadest terms, the organic components include small molecules, polymers, biomacromolecules, as well as bacteria, viruses, and cells (including their membranes and organelles). Tables 1 and 2 show partial lists of inorganic and organic components that have been hybridized for biomedical applications. For each class of hybrid nanomaterials, it is worth noting their vast diversity in terms of composition, structure, and morphology. The two components to be chosen for hybridization are determined by the application of interest.

In addition to the conventional Scheme of hybridization, materials in the same category but with distinct properties have also been integrated into a hybrid system to achieve the multifunctionality sought for various biomedical applications. Notable examples include the development of agents for combination therapy,^[77]^ theranostic agents for both diagnosis and treatment,^[78]^ and contrast agents capable of working with multiple imaging modalities.^[79]^ In preparing this article, we aim to cover both hybrid systems, albeit with a focus on the first type that involves inorganic and organic components. Specifically, we first discuss the necessity to hybridize different types of materials in meeting the demands from a spectrum of biomedical applications. We then review the methods or techniques that have been developed for the fabrication of hybrid nanomaterials. Throughout the discussion, we emphasize the concept of rational design when developing hybrid nanomaterials to meet the requirements of the intended applications. Afterwards, we use various examples to illustrate how hybrid nanomaterials have advanced biomedical research in the context of nanomedicine, regenerative medicine, biomimetics, and biological manipulation.

## 2. Fabrication of Hybrid Nanomaterials

**Figure 2.**
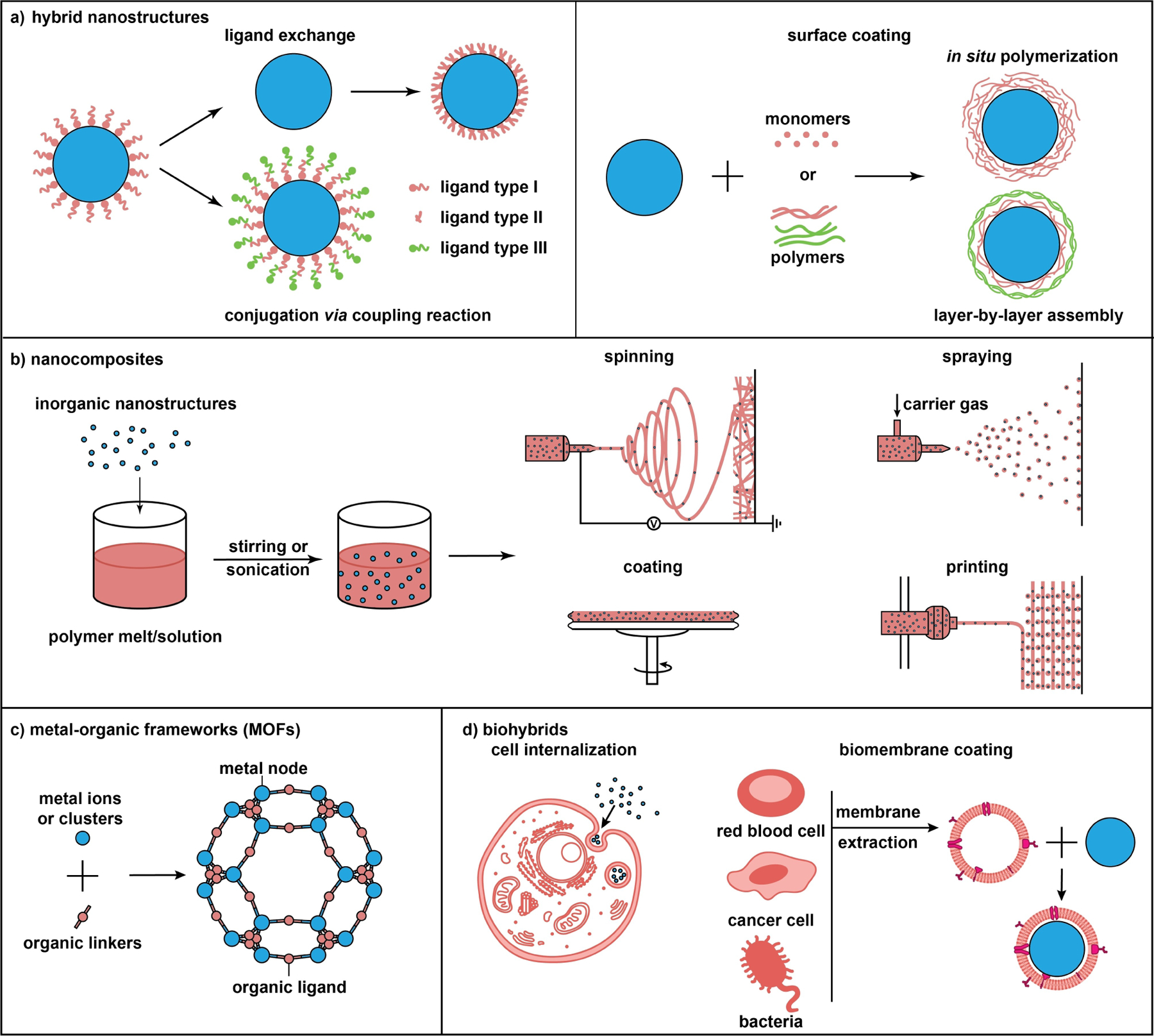
Methods for fabricating hybrid nanomaterials, including a) ligand exchange, conjugation via coupling reaction, and surface coating for hybrid nanostructures; b) mixing or blending for nanocomposites, followed by spray, spinning, coating, and printing to create various morphologies; c) synthesis of MOFs, and d) fabrication of biohybrids.

As illustrated in Figure 2, different forms of hybrid nanomaterials call for different fabrication methods. In this section, we provide a brief introduction to the typical methods, with a focus on the general principle, compatibility between inorganic and organic components, yield, technical limitations, and possible extensions to different materials and systems.

### 2.1. Hybrid Nanostructures

For hybrid nanostructures, the inorganic components are typically prepared with nanometer sizes using a bottom-up or top-down approach, followed by the addition of an organic component to the surface in a process also known as surface modification, conjugation, grafting, or coating. During bottom-up synthesis, a colloidal stabilizer (small molecule or polymer) must be included to prevent the nanostructures from agglomeration.^[80]^ The stabilizer may also act as a capping agent to help control the shape evolution. The product is actually a hybrid material albeit most studies reported in literature only paid attention to the inorganic component. In essence, whenever an inorganic nanomaterial is applied to a bio-related study, one genuinely deals with a hybrid system due to the presence of, at least, an organic stabilizer on the surface. For example, the surface of AuNPs prepared using the Turkvich method is covered by citrate ions, which are used as a reductant for the Au^III^ precursor.^[81]^ For Ag nanostructures prepared using the polyol method, their surface is covered by PVP,^[82]^ a popular colloidal stabilizer for the preparation of metal nanomaterials.

If the organic stabilizer acquired from synthesis is not optimal for the intended application, it can be substituted with the desired one via an exchange process.^[83]^ Depending on the properties of the existing and incoming ligands, the exchange must be conducted with caution to avoid agglomeration. In general, the incoming ligand should have a stronger affinity to the surface of the inorganic component than the existing one. For the identification of a proper ligand, one can refer to numerous review articles on SAMs.^[84]^ Sometimes, additional steps, such as the deposition and then removal of an ultrathin shell of Ag, can facilitate ligand exchange.^[85]^ Many surface ligands can be dedicatedly introduced, including organic molecules with specific charges, adjustable hydrophilicity, or ability to complex with metal ions; synthetic polymers; and biological molecules or macromolecules (e.g., amino acids, peptides, oligonucleotides, proteins, and antibodies) to ensure biocompatibility. As a notable example, the surface of nanostructures is often grafted with polymer brushes based on PEG and its derivatives to provide anti-fouling capability.^[86]^ The PEGylated nanostructures can be readily tailored by varying the molecular weight, architecture, and terminal group. Alternatively, polymers such as zwitterionic polyelectrolytes, polyacrylamides, polyacrylates, Pluronics, and polysaccharides have been explored.^[87]^ Ligand exchange also offers other features or capabilities such as the integration of diverse functionalities by conjugating different ligands to the same nanoparticle.^[88]^ Despite the advancement in analytical methods, it remains a challenge to quantify the extent of ligand exchange.

**Figure 3.**
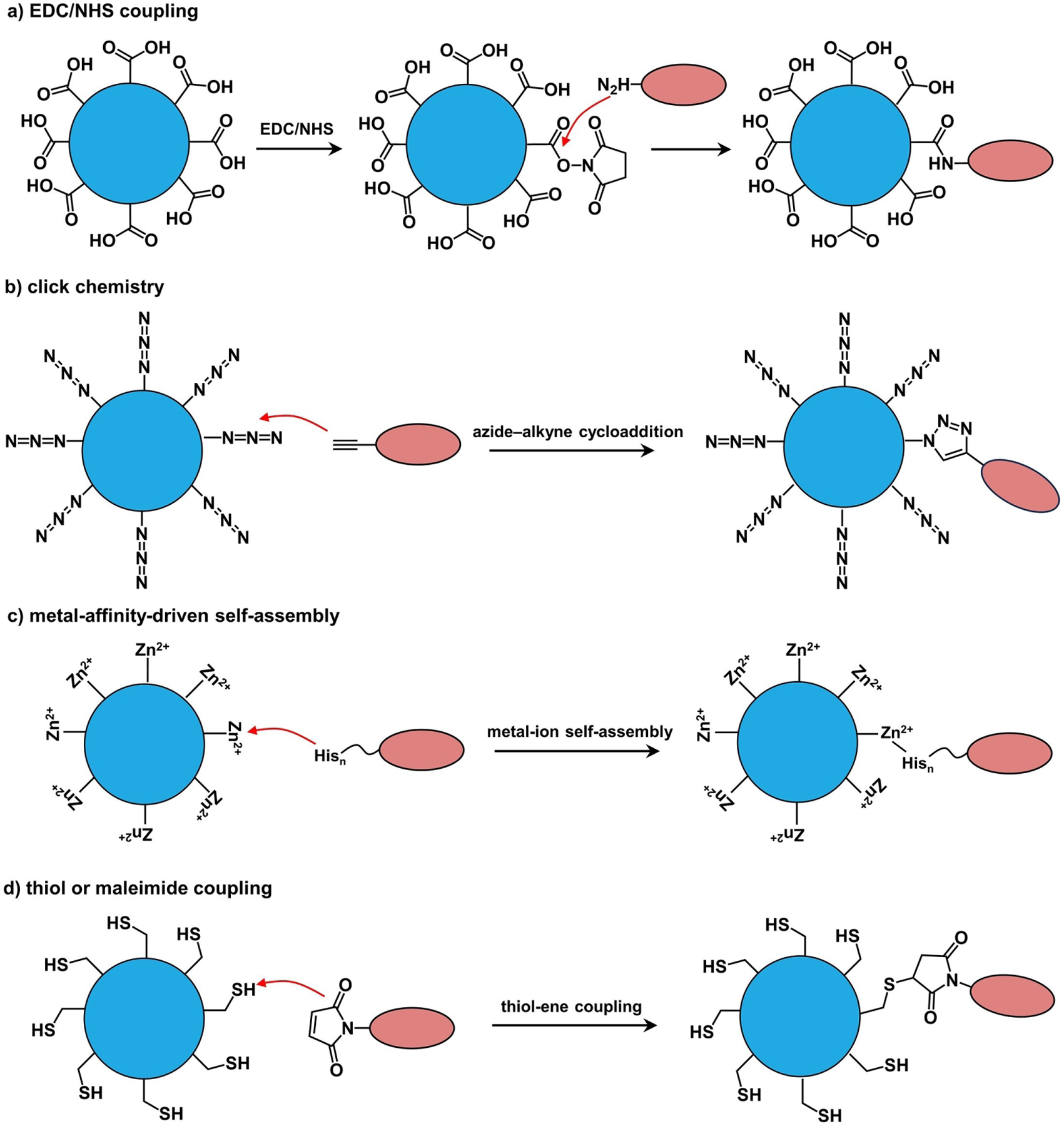
Schematics showing conjugation between inorganic and organic components through a) EDC/NHS coupling, b) click chemistry, c) metal-affinity-driven self-assembly, and d) thiol or maleimide coupling.

Besides exchange, organic components can be covalently conjugated to the existing ligands via coupling reactions. Figure 3 shows some typical examples, including *i*) 1-ethyl-3-(3-dimethylaminopropyl)carbodiimide/N-hydroxy succinimide) (EDC/NHS) coupling,^[89]^ as exemplified by the sulfo-NHS coupling between the carboxyl group on a nanostructure and the amine group on a biomolecule, and *vice versa*;^[90]^ *ii*) click chemistry (or azide–alkyne Huisgen cycloaddition),^[91]^ which requires biomolecules pre-modified with azide or alkynes, together with azide- or alkyne-functionalized nanostructures; *iii*) metal-affinity-driven assembly between polyhistidine-appended peptides/proteins and metal-rich nanostructures,^[92]^ and this strategy relies on the affinity between polyhistidine tags and transition metal ions such as Zn^2+^; and *iv*) thiol-maleimide coupling, which starts with the nanostructures pre-modified with cysteine or sulfhydryl groups that conjugate with cysteine- or sulfhydryl-containing peptides and proteins.^[93]^

A thick organic shell can be formed around an inorganic component to generate a core–shell structure through methods such as atom transfer radical polymerization (ATRP) or layer-by-layer self-assembly.^[94]^ The thick shell can serve as a physical barrier to minimize water interaction with the inorganic surface and thus retard the possible release of toxic ions into the biological system. Depending on the protocol, the product may take a concentric, non-concentric, or Janus structure. In some cases, multiple layers of coating are added to the surface of inorganic nanostructures to further enhance or enrich their properties.^[95]^ For instance, magnetic nanoparticles are commonly coated with SiO_2_, carbon, or Au before being functionalized with an organic ligand to maximize their stability and biocompatibility. A hybrid, multilayered magnetic core–shell system was also developed for the controlled release of paclitaxel.^[96]^

### 2.2. Nanocomposites

Nanocomposites refer to hybrid materials comprised of inorganic nanostructures dispersed in polymer matrices.^[97]^ They can be produced by mixing the dry powders or a colloidal suspension of the inorganic component with a polymer solution, followed by solvent removal via evaporation^[39]^ or freeze drying.^[98]^ Alternatively, melt mixing can be used for systems involving a thermoplastic matrix.^[99]^ In this case, the polymer matrix is softened at an elevated temperature and then blended with the inorganic component. Melt mixing has the advantage of being compatible with many conventional polymer processing techniques while not requiring an organic solvent,^[100]^ albeit it only works at a low loading of the nanostructures. Upon adding the inorganic component, the viscosity of the mixture quickly rises, rendering further mixing infeasible. During mixing, stirring or sonification is typically used to achieve homogenization. The nanocomposites tend to suffer from the heterogeneity caused by agglomeration, which may compromise their properties and performance. Due to the high specific surface energy intrinsic to a nanosystem,^[101]^ it is vital to obtain a uniform mixture by optimizing the inorganic/organic interface while augmenting the adhesion between the two components. This is often achieved by adding a specific functional group to the surface of the nanostructures via covalent coupling or non-covalent adsorption.^[102]^

Alternatively, methods involving in situ polymerization and/or formation of nanostructures have been developed to obtain nanocomposites with improved homogeneity and even allow for a tight control over the distribution of inorganic nanostructures in polymer matrix.^[103]^ Typically, in situ polymerization is conducted by mixing the inorganic component with a liquid monomer, followed by polymerization (or cross-linking) to solidify the mixture. This process can be triggered using an external stimulus such as heating or radiation and is facilitated through the addition of an initiator, crosslinker, and/or catalyst.^[104]^ For example, a nanocomposite comprised of graphene and hydrogel was fabricated by initiating gelation with peroxide immobilized on the graphene.^[105]^ The inorganic nanostructures can also be formed in situ through the reduction of a metal precursor, the use of sol-gel chemistry, or a hydrothermal process.^[106]^ Leveraging on the protocols for the colloidal synthesis of inorganic nanostructures, this approach not only prevents their agglomeration in the polymer matrix but also enables better control over their size and shape, enhancing the tunability of the nanocomposite. As a drawback, the residual reactants may cause unpredictable alternations to the properties.^[107]^

The properties of nanocomposites can be engineered by controlling the filling ratio and/or spatial distribution of the fillers. If the fillers are incorporated to engineer mechanical properties, changes in filling ratio typically have opposing effects on the tensile strength and elasticity. While tensile strength increases as the filling ratio increases due to the presence of more nanostructures inside the polymer matrix, the elasticity decreases because of the reduction in free volume for the matrix.^[108]^ If the filling ratio is too high, however, it would cause agglomeration, decreasing the tensile strength.^[109]^

**Figure 4.**
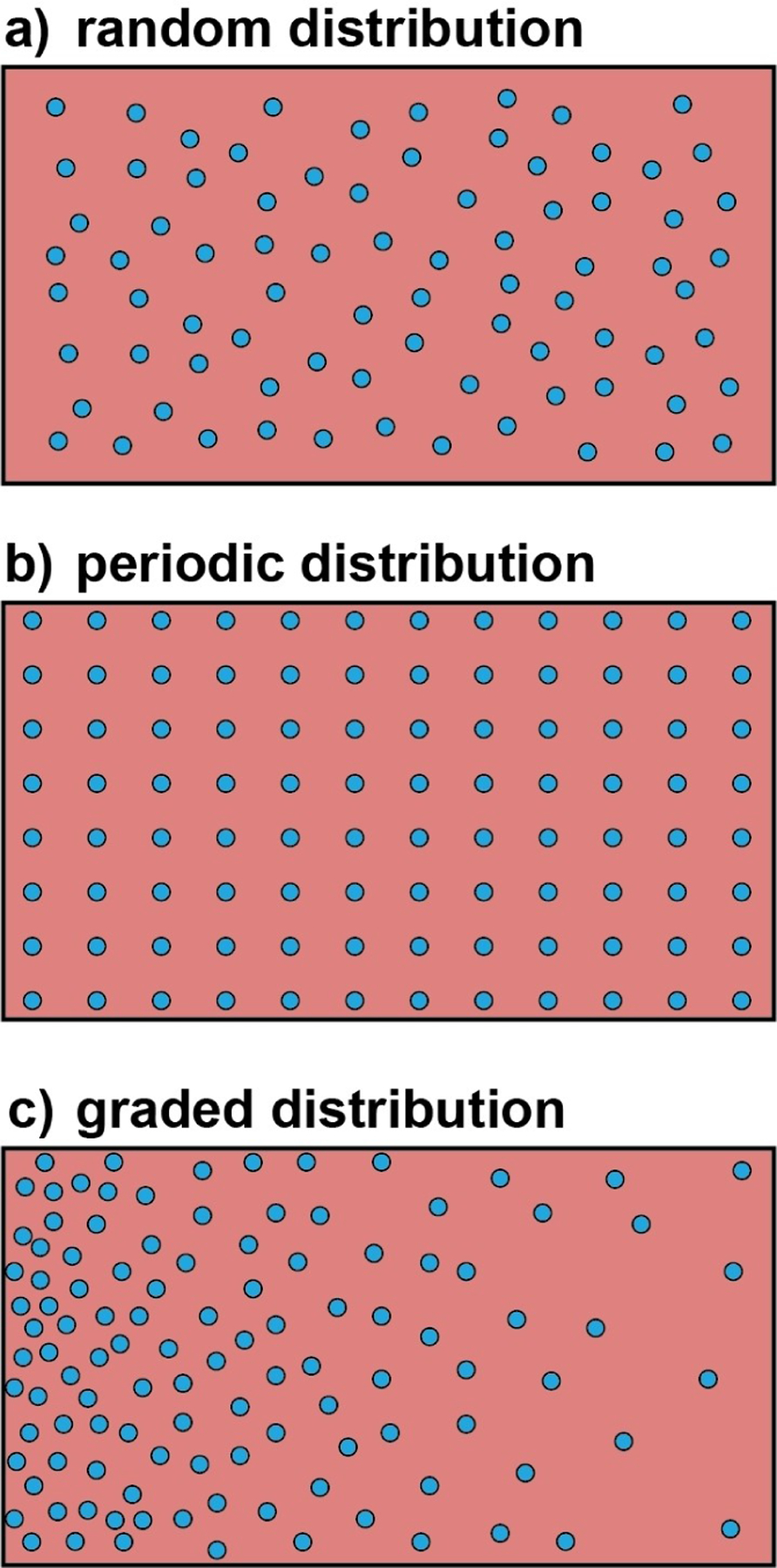
Schematics of nanocomposites in which the inorganic component takes a) random, b) periodic, and c) graded distributions, respectively.

In general, the inorganic nanostructures are randomly distributed in the polymer matrix to yield a homogeneous, isotropic structure (Figure 4a). To better suit some biomedical applications, nanocomposites with specific arrangements for the fillers have been fabricated. In one study, the template-assisted formation of a polymer matrix was used to obtain nanocomposites featuring a periodic array of inorganic components (Figure 4b).^[110]^ Specifically, inorganic nanostructures with self-assembly capability are used as templates and annealed to fix their periodic arrangement. An organic phase then infiltrates into the ordered network, forming the dispersion phase. In addition to the high degree of uniformity throughout the material, the nanocomposites fabricated using this method have unique photonic and electrical properties for bioelectronic applications. Serving as biomimetic scaffolds, nanocomposites with graded distributions of inorganic phase are preferred for the repair of tendon-to-bone enthesis as they can recapitulate the features of a native structure (Figure 4c). Techniques like force-driven sedimentation, diffusion,^[39,111]^ and layer-by-layer coating^[112]^ are used to produce this type of nanocomposite.

The properties of nanocomposite also depend on the composition, shape, and morphology of the filler. Many inorganic fillers, including metal nanoparticles,^[103]^ ceramic nanostructures,^[113]^ and carbon nanomaterials,^[114]^ have been explored to bring in unique functions. While most of the fillers enhance mechanical properties, those based on carbon nanomaterials or metal nanowires are effective in endowing the product with superior electrical conductivity. Others, like HAp, are bioactive and can naturally interact with the biological system once implanted. For fillers of the same composition, their enhancement effect on the nanocomposites typically depends on the shape or morphology. It was reported that multi-walled CNTs were more effective than graphene nanoribbons in improving electrical conductivity due to the enhanced ability to interlace with each other.^[41]^

Nanocomposites can be processed into 1D, 2D, or 3D structures using various techniques. For example, a polymer solution or melt containing a precursor or preformed nanostructures can be extruded using wet-, melt-, or electrospinning to produce 1D structures such as fibers.^[115]^ The spinnability is primarily determined by the nature of the polymer matrix and the viscoelastic properties of the solution or melt and it also depends on the precursor, as well as the size, shape, and concentration of the nanostructures.^[116]^ Compared with wet- and melt-spinning that produce micron-to-millimeter fibers, electrospinning utilizes electrostatic repulsion to generate ultrathin fibers down to the nanoscale regime.^[116,117]^ The nanofibers are not only on a scale similar to that of collagen fibers in ECM,^[118]^ but also mimic the elongated morphology of tissues such as tendons and nerves,^[119]^ making them excellent scaffolding materials. The structure and morphology of the nanofibers can be tuned by adjusting the physical properties (e.g., inorganic content, viscosity, and solution conductivity) of the materials and the processing parameters such as ejection rate, voltage, and collector distance. By manipulating the charge, orientation, or motion of the collector, the nanofibers can be collected as 2D mats with distinctive patterns.

Another representative 2D morphology adopted by nanocomposites is thin films, which are typically fabricated using solution casting,^[39]^ brush coating,^[112]^ and spin coating.^[120]^ Among them, spin coating is widely utilized due to its ability to produce uniform films with high batch-to-batch consistency. In a typical process, a nanocomposite suspension is deposited on a static substrate that is then accelerated to spin at a high speed, spreading out the material into a thin film by centrifugal force. While the rheology of the liquid dictates its behavior during the spin process, the thickness and the uniformity of the resultant film are correlated with the acceleration and angular velocity of the spin. By applying solutions with decreasing or increasing inorganic loadings, one can generate thin films with a gradient in mineral content.^[121]^ Recently, wet- and electrospraying have received increasing attention as a substitute of spin coating for the fabrication of nanocomposite films on larger and nonplanar substrates.^[122]^ When subjected to a physical or electrostatic force, numerous fine droplets are ejected and deposited onto the substrate, followed by their coalescing into a thin film upon solvent evaporation. Material feeding rate, applied pressure, and collection distance are all important in determining the outcome.

Although 2D films of nanocomposites can be stacked in a layer-by-layer fashion to create a 3D structure, the variations attained using this method are limited. Additive manufacturing, also known as 3D printing, is increasingly used to process nanocomposites into complex 3D structures, including personalized cardiovascular implants and orthopedic prosthetics.^[123]^ Like the spinning techniques, the 3D printing of nanocomposites also faces the challenge of balancing the printability of the material and its ultimate properties. Although some researchers have demonstrated 3D printing of nanocomposites based on hydrogels^[124]^ and PLGA^[125]^ for antimicrobial and scaffolding applications, much work remains to be done to improve the printability of the ink and stabilize the nanocomposites during the printing process to increase the reproducibility and expand their scope of application.^[126]^

### 2.3. MOFs

Characterized by an extended inorganic-organic coordination network, MOFs are comprised of a proper combination of metal centers (ions, clusters, or multinuclear complexes) connected by di- or polydentate organic linkers.^[127]^ The highly open structure endows MOFs with unique properties, including high porosity, low densities (0.2–1 g/cm^3^), and huge specific surface areas (500–4500 m^2^/g), enabling them to adsorb a large number of small molecules.^[128]^ When applied to biomedical applications, the external surface of MOFs must undergo modification (see section 2.1) to acquire the proper functional group(s) to interact with the biological system of interest.

**Figure 5.**
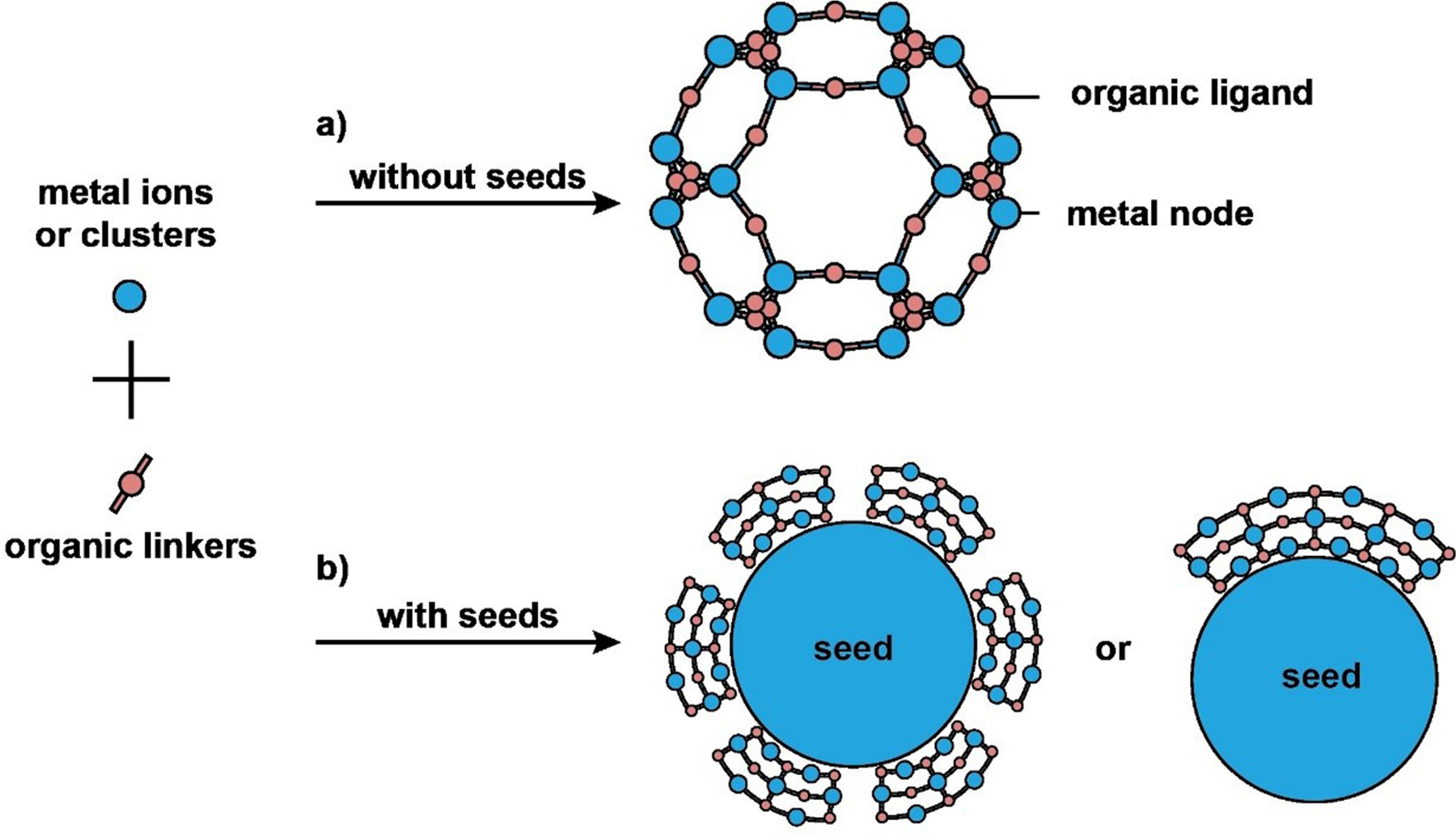
Schematic showing two methods for the synthesis of nanostructured MOFs, in the absence and presence of seeds, respectively.

A variety of methods, including those based on solvo(hydro)thermal, ultrasonic, microwave-assisted, reverse microemulsion, mechanochemical, and electrochemical techniques, have been developed to synthesize MOFs.^[129]^ The methods can be divided into two categories depending on the absence or presence of seeds. Most MOFs are synthesized using a one-pot, seed-free protocol. In this case, nuclei are formed in a nearly supersaturated solution through homogeneous nucleation that involves the self-assembly of metal ions and ligands, followed by splitting or recombination of primary nuclei in the initial growth step.^[130]^ These dynamic processes then reach an equilibrium, yielding a large number of seeds that can evolve into MOFs (Figure 5a).^[131]^ For example, Gd-based MOFs were synthesized using a reverse microemulsion system in which nanoscale MOFs grew from a microemulsion comprised of GdCl_3_ precursor, cetyltrimethylammonium bromide (CTAB), heptane, 1-hexanol, and water.^[132]^ To stabilize the Gd-based MOFs and enhance their biocompatibility, a tertiary amine-based pH-responsive copolymer (e.g., PEG-*b*-PDPAEMA) was synthesized by reversible addition–fragmentation chain transfer (RAFT) polymerization and then grafted to the surface. The polymer-coated, Gd-based MOFs are biocompatible and capable of MRI and cancer targeting.

The other synthetic strategy involves heterogeneous nucleation of MOFs on the surface of existing nanoparticles with matched crystal lattices. This method is referred to as seed-mediated growth (Figure 5b).^[133]^ In general, heterogeneous nucleation requires a much lower driving force than does the homogeneous process. The introduction of seeds made of a different material also facilitates the synthesis of hybrid nanostructures. In one study, Zr-porphyrinic MOFs were grown on the surfaces of a variety of organic and inorganic nanostructures, including PDA nanoparticles, AuNRs, and GO sheets.^[65]^ The success relies on the ability to control the coordination interactions between the functional groups on the seed and the Zr metal nodes, which not only facilitated heterogeneous nucleation but also allowed for a tight control over the thickness of the MOFs. The hybrid nanostructures could be directly applied to combination cancer treatment due to the photodynamic activity of porphyrin and the photothermal effect of PDA. Using a similar strategy, MOFs were grown on PVP-covered lanthanide-doped UCNPs to obtain UCNP/MOF heterodimers with an asymmetric distribution of compositions.^[134]^ The heterodimers allowed for efficient photon harvesting through the resonance energy transfer from UCNP to MOFs, facilitating the production of singlet O_2_ under NIR irradiation. This hybrid system also offers a promising platform to combine NIR-induced photodynamic therapy with photothermal therapy and chemotherapy for effective cancer treatment.

Stability has been a major concern of MOFs. Despite escalated research in recent years, MOFs still lag behind other porous materials like zeolites, mesoporous silica, and porous carbons in terms of robustness. Additionally, there is still no tight control over the size and shape of nanostructured MOFs. Although exotic seeds offer better control on these parameters, their removal may involve time-consuming, environmentally unfriendly processes, hindering scale-up production. These two issues need to be addressed before MOFs can be fully applied to biomedical applications.

### 2.4. Biohybrids

Inorganic nanostructures can be loaded into biological systems such as cells, bacteria, organelles, and membrane vesicles to obtain biohybrids. Such hybridization offers a natural vehicle to integrate the physical (e.g., optical, electrical, and magnetic) and chemical (e.g., reactivity and catalytic) properties typical of inorganic nanostructures with the biocompatibility, immune evasion, and transmembrane transport attributes intrinsic to biological systems. Depending on the type and source of the biological component, the fabrication typically involves cellular internalization and wrapping with a natural biomembrane.

**Figure 6.**
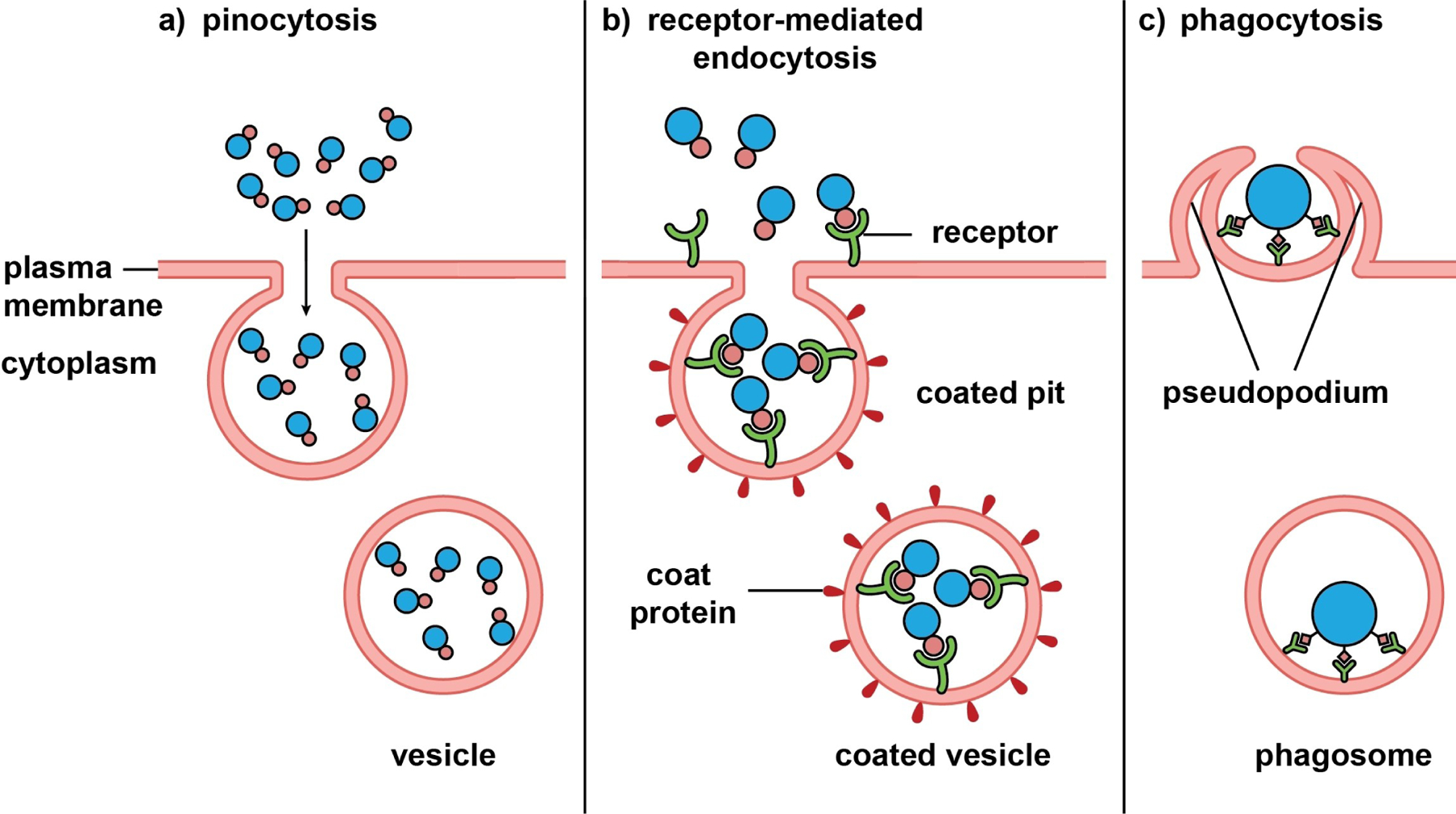
Schematics showing different approaches to the fabrication of biohybrids through cellular internalization of inorganic nanostructures.

Cellular internalization has been widely explored for drug delivery, imaging, cancer therapy, and tissue repair.^[135]^ As a living organic component, the cell offers smart, adaptive, and dynamic characteristics.^[136]^ The internalization process may involve different pathways such as pinocytosis, micropinocytosis, clathrin- or caveolae-mediated endocytosis, and phagocytosis, as illustrated in Figure 6.^[137]^ Upon internalization, the nanostructure is typically trapped in the endosome, transferred to the lysosome, and degraded or expelled into ECM, while it can also escape from the endosome and enter the cytosol or other cellular organelles such as mitochondria and even the nucleus.^[138]^ The exact pathway is determined by the cell type and the properties of the nanostructure, including size, shape, morphology, surface charge, and surface functional group.

Noble-metal nanostructures have been actively explored to fabricate biohybrids via cellular internalization for applications related to cancer theranostics, known as the Trojan horse strategy. In particular, owing to their controllable sizes, diverse surface ligands, bio-inertness, and good biocompatibility, Au-based nanostructures (e.g., nanoparticles, nanorods, and nanocages) have received the most attention. In addition to their roles as contrast agents for tracking through various imaging modalities, the nanostructures offer therapeutic capabilities through photothermal heating and/or drug loading. Other functional nanomaterials such as Fe_3_O_4_ nanoparticles have received attention for MRI-guided therapy.^[139]^ In essence, the unique inorganic-biological hybridization strategy can combine inorganic nanostructures with living organic components to make them work synergistically.

**Figure 7.**
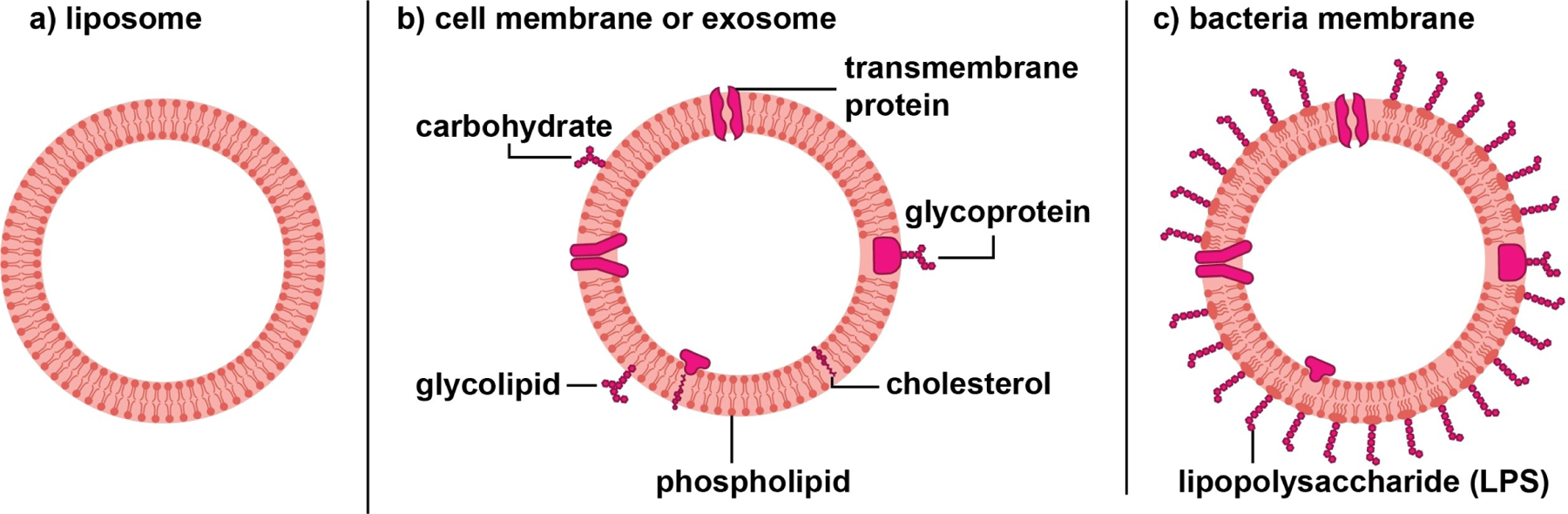
Schematics of different types of biomembranes, including a) liposome, b) cell membrane or exosome, and c) bacteria membrane.

Instead of using whole cells, their membranes can also be directly utilized to encase inorganic nanostructures and obtain biohybrids (Figure 7). Due to the good stability, low immunogenicity, and low toxicity of natural biomembranes, the biohybrids exhibit excellent biocompatibility, multifunctionality, and targeting capability. The biomembranes can come from a broad range of natural sources, including cell membranes, liposomes, exosomes, and bacterial membranes, corresponding to different hybridization methods.^[140]^

In the first method, membranes are extracted from various types of cells, including red blood cells, cancer cells, macrophages, and mesenchymal stem cells, and directly utilized to encapsulate inorganic nanostructures.^[140]^ Through a series of steps involving sonication, extrusion, and differential ultracentrifugation, the cell membranes are isolated as vehicles, into which inorganic nanostructures are introduced via sonication, electroporation, or fusion in a microfluidic device to generate biohybrids.^[139]^ Owing to the diverse sources of cell membranes, the biohybrids can be fabricated with unique biomimetic capabilities. For instance, the biohybrids comprised of cancer cell membranes possess the specific antigens from homologous cancer cells to activate cancer immunotherapy,^[141]^ whereas the modification involving red blood cell membranes can improve the transepithelial transport and blood circulation capabilities.^[142]^

The second method involves liposomes comprised of a lipid bilayer encapsulating a water core, with sizes ranging from 100 nm to 2.5 mm. Owing to the amphiphilic nature of lipids, the core of a liposome can be loaded with hydrophilic molecules, while the lipid bilayer can serve as a carrier for hydrophobic species. The molecular loadings of liposomes can be further integrated with inorganic nanostructures to create highly efficacious therapeutic functions. For instance, utilizing liposome’s acid-responsive and fusion activity, the biohybrid comprised of carboxyl-terminated AuNPs in phospholipid liposomes holds the promise to treat various skin diseases such as staph infections.^[143]^

The third method is based on exosomes, extracellular vesicles with sizes ranging from 30–150 nm.^[144]^ Different from liposomes, exosomes possess functional membrane proteins and can act as a crucial mediator for intercellular interaction and targeted delivery. The extracted exosomes can encapsulate inorganic nanostructures by electroporation or microemulsion methods. Serving as a protective shell, the unique biomimetic structure of exosomes enables the biohybrid to evade clearance in vivo, offering high targeting efficiency. In one example, the biohybrid constructed from iron oxide nanoparticles promotes M1 macrophage polarization, enhancing immunity against hepatocellular carcinoma.^[145]^

Owing to the simple structure and composition, rapid growth speed, and ease of cultivation, the biomembranes from bacteria have also been exploited to modify inorganic nanostructures for the fabrication of biohybrids. In particular, the vesicles secreted by gram-negative bacteria are a good source of biomembranes for activating pathogen-related innate and adaptive immune responses.^[146]^ On the other hand, the S-layer protein of bacteria, along with the negative surface charge, makes it possible to recruit inorganic metal ions for the creation of nucleation sites. In particular, the biohybrid derived from bacteria-mimetic AuNPs can be selectively phagocytosed by phagocytic immune cells to create biohybrids capable of targeting melanoma via inflammatory tropism. The photothermal and immune responses could work synergistically to eradicate cancer cells.^[147]^

In addition to the methods noted above, other strategies such as bioconjugation have been explored to construct biohybrids. Different bioconjugation strategies, including carboxylic acid-amine reaction, click chemistry, and thiolene reaction, have been developed to help preserve the activity of specific biomolecules.^[148]^ For example, by conjugating AuNPs with the lipids of living cell membranes, one can preserve the sensing and photothermal capabilities of AuNPs, providing a tool for cell tracking and local environment sensing.^[149]^ Built upon the fabrication methods noted above, other kinds of inorganic–inorganic, inorganic-organic, and organic-organic hybrid nanostructures or nanocomposites can be further hybridized with various biological components to fabricate flexible, stable, and multifunctional bioconstructs.

The field of biohybrids still faces several challenges. First of all, it is critical to ensure that the inorganic nanostructures do not trigger adverse cellular responses in the construction of biohybrids. Many researchers have evaluated biocompatibility, particularly cytotoxicity, in vitro, but it is their behavior and biological impacts in vivo that play a vital role in applying biohybrids to clinically relevant scenarios. Due to the complexity of biological systems, maintaining both the structural stability and the functionality of biohybrids in a physiological environment presents a significant challenge. At the current stage of development, the translation of biohybrids from laboratory to clinical setting has been hindered by the labor-intensive processes, the requirement of specialized equipment, and the lack of reproducibility. We envision that these challenges will be addressed with the advancements in theoretical modeling, fabrication techniques, and characterization methods, unlocking the full promise of biohybrids in various biomedical applications.

## 3. Examples of Hybrid Nanomaterials and their Biomedical Applications

As summarized in Tables 1 and 2, a vast number of hybrid nanomaterials have been explored to advance biomedicine. Here we focus on several selected examples. For each example, we strive to *i*) describe the hybrid system briefly; *ii*) explain why the specific inorganic and organic components are chosen for the target application; *iii*) highlight the merits of material hybridization and the performance of the hybrid system; and *iv*) discuss the possible extensions to other materials, systems, and applications.

### 3.1. Hybrid Nanostructures

We use a set of examples to illustrate how inorganic nanostructures are hybridized with a variety of organic components to enhance and enrich their applications in biomedicine, including targeted delivery and controlled release for nanomedicine, multiplex assay for diagnostics, and fabrication of flexible bioelectronics and nanomotors for healthcare, as well as development of multi-modal imaging contrast agents and theranostic agents for image-guided therapy.

#### 3.1.1. Surface Modification with Organic Ligands for Targeted Delivery

Surface modification plays a vital role in the preparation and utilization of essentially all types of inorganic nanostructures. To this end, organic molecules, biomolecules, biomacromolecules, and polymers (both natural and synthetic) have all been explored as ligands to modify the surface of inorganic nanostructures. They can prevent the nanostructures from aggregation in a liquid while facilitating their interactions with the biological system of interest to optimize pharmacokinetics and biodistribution. By modifying the surface of inorganic nanostructures with proper ligands, reduced immunogenicity and opsonization,^[150]^ increased circulation time,^[151]^ and targeted delivery^[18]^ have all been accomplished.

PEGylation, which refers to surface modification with PEG, is one of the most commonly used strategies for improving blood circulation and biodistribution. It is proven that PEGylation can shield manmade nanomaterials from the mononuclear phagocytic system (MPS) by increasing surface hydrophilicity and decreasing protein adsorption.^[152]^ For example, the blood circulation half-life of mesoporous silica was increased by 200 times through PEGylation.^[153]^ PEGylation is also effective in delaying the clearance of organic nanoparticles. It was reported that PEGylation greatly reduced the accumulation of PLGA or PCL nanoparticles in the liver.^[154]^

**Figure 8.**
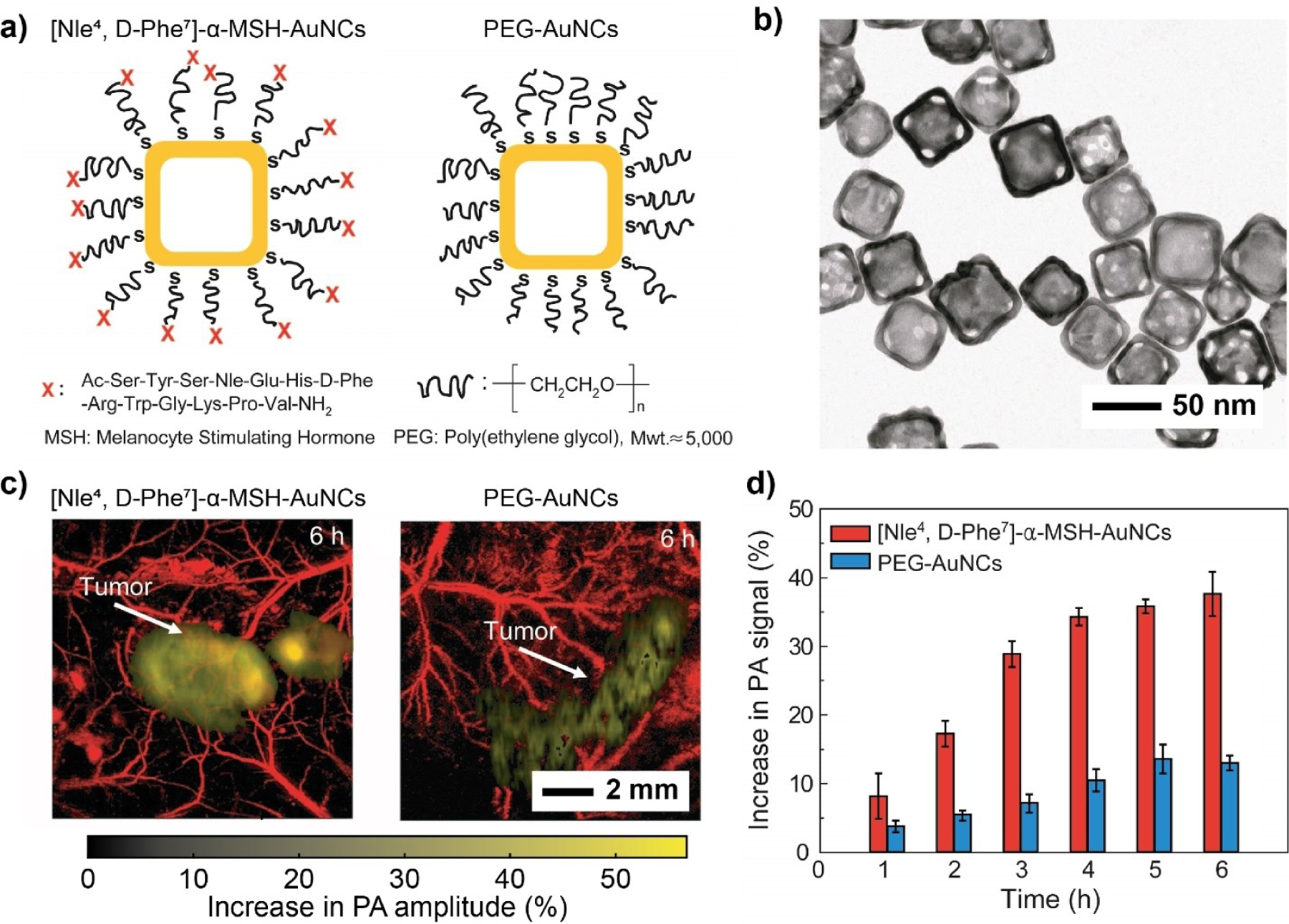
a) Schematic of [Nle^4^,d-Phe^7^]-α-MSH-AuNCs (left) and PEG-AuNCs (right) to be evaluated for active and passive targeting, respectively. b) Transmission electron microscopy (TEM) image of a typical sample of AuNCs. c) PA images, with yellow and red colors corresponding to the melanoma and the tumor-feeding blood vessels, respectively. d) Changes in PA amplitude after intravenous injection of [Nle^4^,d-Phe^7^]-α-MSH- and PEG-AuNCs (*n* = 4, *p* < 0.0001), respectively. Reproduced with permission from ref. [18]. Copyright 2010 American Chemical Society.

While improved circulation generally increases the availability of a nanomedicine, targeted delivery can take this strategy to another level of success. On top of enhancing the efficacy of the medication, targeted delivery helps minimize off-target toxicity and thereby reduces damage to healthy tissues. By hybridizing with proper ligands, nanostructured materials can be delivered to the malignant site through a passive^[155]^ or active targeting mechanism.^[18,156]^ In one study, PA imaging was used to quantitatively compare the efficacy of passive versus active targeting in a subcutaneous tumor mouse model.^[18]^ Specifically, the surface of AuNCs was modified with a PEG-based ligand terminated in [Nle^4^, d-Phe^7^]-α-melanocyte-stimulating hormone ([Nle^4^, d-Phe^7^]-α-MSH) for targeting melanoma (Figure 8a,b). The AuNCs conjugated with PEG (PEG-AuNCs) served as a control for passive targeting. After intravenous injection of [Nle^4^, d-Phe^7^]-α-MSH- and PEG-AuNCs, PA images were recorded at different intervals of time. The images obtained at *t* = 6 h post-injection showed PA signal enhancement in the melanoma tissue for both groups, with the active targeting group showing a superior result (Figure 8c). For active targeting, the maximum PA signal increased up to 38±6%, almost 300 % greater than the signal increase arising from passive targeting (Figure 8d). The trend was further confirmed by measuring the Au contents in the dissected melanomas. Per tumor mass, the number of [Nle^4^, d-Phe^7^]-α-MSH-AuNCs accumulated in the melanoma was 3.6 times that of PEG-AuNCs.

Many other imaging modalities have also benefited from surface modification with organic ligands. For example, PEGylated Ag_2_S QDs were used for in vivo NIR fluorescence imaging of subcutaneous tumors in mice.^[27]^ Superior MRI performance was also reported for a PEGylated superparamagnetic iron oxide (SPIO)-antibody conjugate.^[32]^ Additionally, ligand-covered Au nanostructures have been exploited for CT,^[157]^ SERS,^[158]^ and other clinically relevant imaging modalities.

Essentially, the surface of all manmade nanomaterials must be functionalized before they can be applied to a biomedical study. Besides PEG and its derivatives, peptides,^[55]^ charged polymers,^[50,51]^ zwitterionic molecules,^[67,159]^ and oligonucleotides^[160]^ are some of the compelling alternatives for surface modification. For instance, Fe_3_O_4_ nanoparticles functionalized with positively charged dendrimers were shown to achieve more efficient cellular uptake compared to the unfunctionalized ones or those with neutral dendrimers.^[51]^ In particular, due to the formation of a stable hydration layer through electrostatic interaction, zwitterionic molecules offer a powerful alternative to PEG in terms of resisting non-specific protein adsorption and anti-fouling.^[67,161]^ Apart from these, oligonucleotide-functionalization has opened the door to new opportunities in nanomedicine. With good targeting ability, oligonucleotides have been conjugated to many types of inorganic nanomaterials–including Au, Si, SiO_2_, and iron oxide nanoparticles–for targeted delivery.^[162]^ In one example, MNSPs loaded with an anti-cancer drug were conjugated with single-stranded oligonucleotides to target breast cancer cells.^[34]^ Compared to the pristine sample, the oligonucleotide-modified ones were more effectively internalized by the cells, leading to increased efficiency in killing cancer cells.

In general, surface modification with molecular ligands applies to many other case scenarios, including sensing and tissue engineering. We will cover some of these in more detail in the following sections.

#### 3.1.2. Hybridization with Organic Materials for Controlled Release

Controlled release pertains to delivery methods capable of gradually discharging the payload over an extended period by upholding a steady and manageable release kinetics.^[163]^ Among various strategies for controlled release, the smart systems involving stimuli-responsive materials have attracted the most attention.^[164]^ Aligned with the ongoing drive to minimize the adverse impacts of a therapeutic agent, stimuli-responsiveness offers a means for on-demand release in response to chemical, physical, or biological cues.^[165]^ From the perspective of material selection, inorganic nanostructures with hollow interiors (pores or cavities) are beneficial to the achievement of high loading capacities.

**Figure 9.**
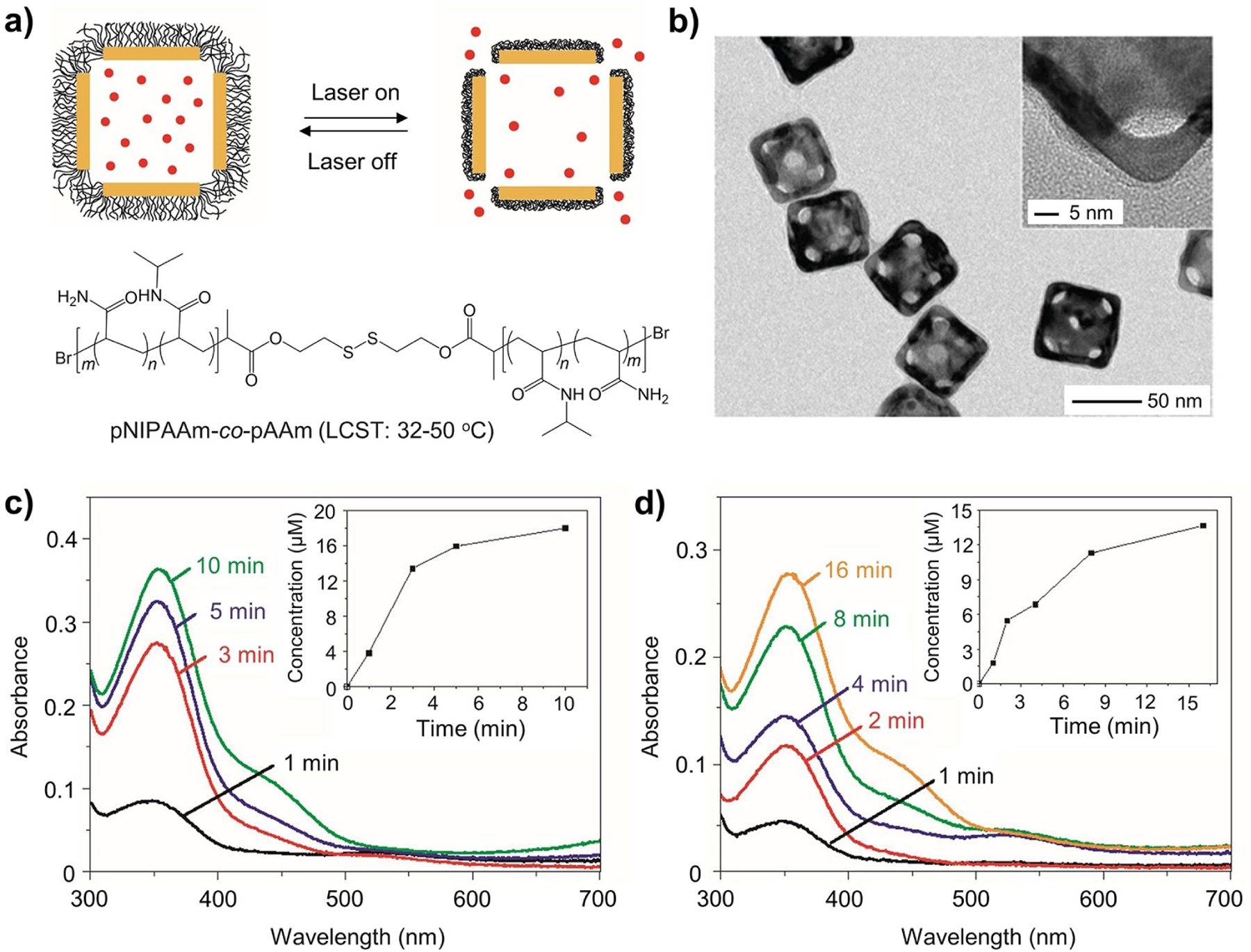
a) Schematic of the hybrid system. Upon exposure to a NIR laser, the smart polymer undergoes a conformational change from extended to collapsed, opening the pores on the surface and releasing the payload. b) TEM images of the AuNCs covered by the thermo-responsive polymer with an LCST at 39 °C. The inset shows a magnified TEM image of the pore region. c,d) Absorption spectra of alizarin-PEG released from the polymer-covered AuNCs by (c) heating at 42 °C for up to 10 min and (d) exposure to a pulsed NIR laser at an irradiance of 10 mW cm^−2^ for up to 16 min. The insets show the cumulative release profiles of alizarin-PEG under the thermal and photothermal conditions. Reproduced with permission from ref. [19]. Copyright 2009 Springer Nature.

Among the external stimuli, light offers potential spatiotemporal controls over the payload release.^[166]^ In one example, a hybrid system was developed by coating AuNCs with polymer brushes made of thermo-responsive pNIPAAm or its copolymers pNIPAAm-*co*-pAAm via the thiolate linkage (Figure 9a,b).^[19]^ When irradiated by a laser at a wavelength overlapping with the absorption peak of the AuNCs, the light is converted to heat through the photothermal effect.^[167]^ In response to the temperature rise, the surface-anchored polymer brushes collapse to open the pores on the AuNCs once the temperature surpasses the lower critical solution temperature (LCST),^[168]^ swiftly releasing the payload. Since the conformational change is reversible, the polymer brushes will revert to the extended state when the laser is turned off, stopping the release. This hybrid system was validated using in vitro assays by monitoring the release of various types of payloads from the AuNCs. Upon irradiation with a pulsed NIR laser, both alizarin-PEG and DOX were quickly released (Figure 9c,d). Following NIR irradiation, a substantial number of breast cancer cells treated with the DOX-loaded AuNCs were eradicated, and the proportion of viable cells decreased as the duration of irradiation was prolonged. As controls, the cells irradiated with the same NIR laser in the absence of AuNCs showed almost no change in viability while the cells treated with AuNCs alone showed a slight reduction in viability due to the photothermal heating.^[169]^ These results demonstrated the feasibility of hybridizing AuNCs with smart polymers to enable NIR-triggered drug release with spatiotemporal controls. In a follow-up study, the same hybrid system was used for controlled release with high-intensity focused ultrasound (HIFU) to improve the penetration depth.^[170]^

**Figure 10.**
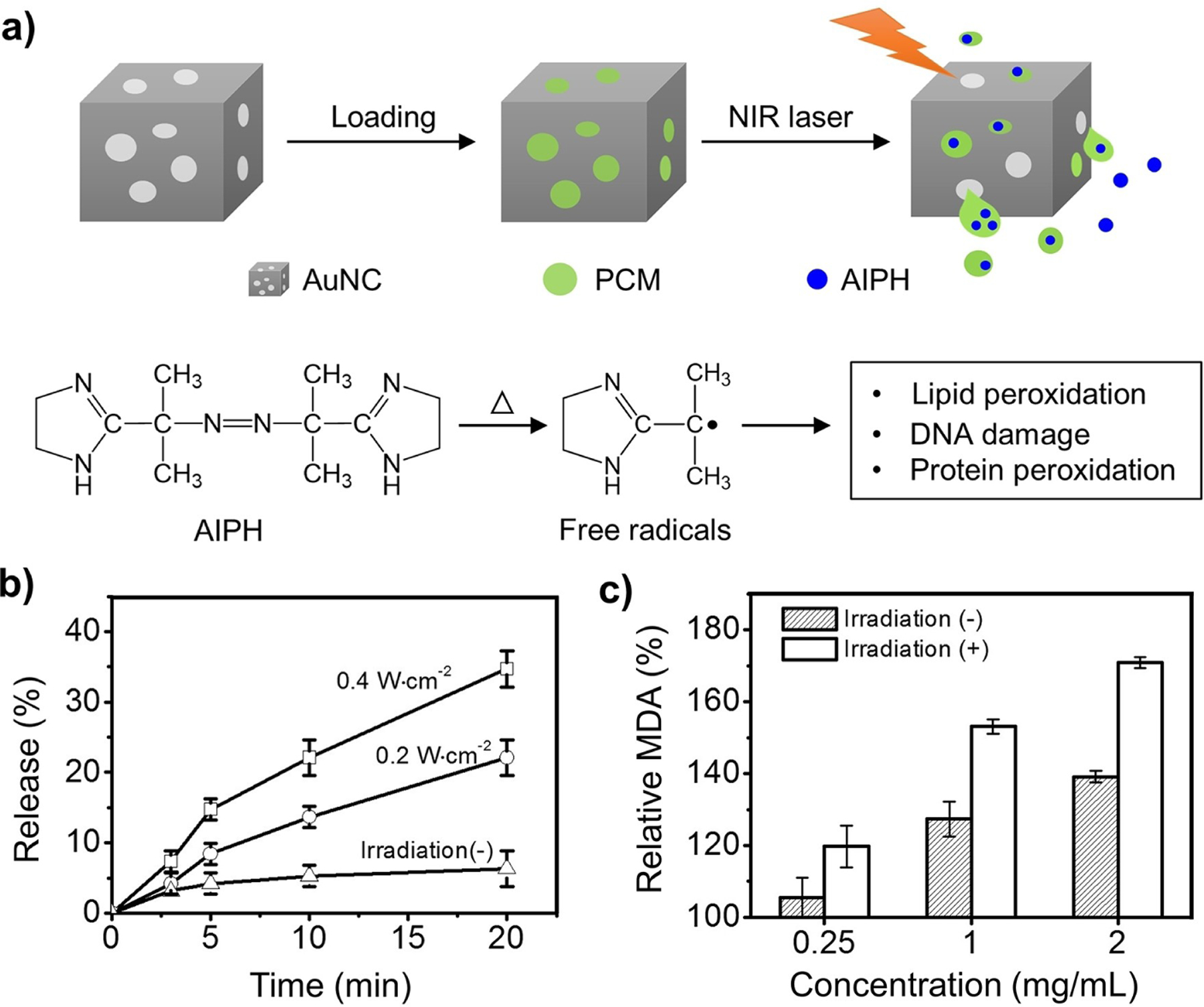
a) Schematic showing the controlled release of AIPH from a PCM-filled AuNC. Due to photothermal heating, the AIPH is further decomposed to generate radicals. b) Release profiles of AIPH from the AuNCs upon NIR irradiation at different irradiances. c) The levels of MDA in RBCs when subjected to AIPH-PCM-AuNCs at varying concentrations in the presence or absence of NIR irradiation. Reproduced with permission from ref. [61]. Copyright 2017 Wiley-VCH.

Apart from attaching polymer brushes to the external surface of inorganic nanostructures, filling the hollow interior with a mixture of the payload and an organic PCM can also be used for controlled release.^[171]^ In one demonstration, the cavities of AuNCs were filled with AIPH and a PCM based on lauric acid (with a melting point of 44–46 °C) to achieve the controlled generation of free radicals for cancer treatment under hypoxia condition (Figure 10a).^[61]^ The radicals interact with various cellular components, inducing cell apoptosis by causing DNA damage, as well as protein and lipid peroxidation.^[172]^ Specifically, upon exposure to a NIR laser, heat was produced owing to the photothermal effect of the AuNCs, melting the PCM and causing the release of the encapsulated AIPH and simultaneous decomposition to generate free radicals. The amount of radicals showed a positive correlation with the irradiance of the laser (Figure 10b). Figure 10c shows the levels of methane dicarboxylic aldehyde (MDA), a byproduct of lipid peroxidation, in red blood cells (RBCs) incubated with AIPH-PCM-AuNCs at different concentrations. This result underscores the creation of radicals under NIR irradiation in the absence of oxygen, promising on-demand release and cancer therapy in both oxygen-rich and deficient environments. The same strategy for controlled release has been extended to other types of hollow nanostructures, including nanobottles made of SiO_2_ and PDA.^[164,173,174]^

Hybridization offers a viable platform for the integration of different materials or components for the achievement of on-demand release. In general, the organic component can be designed to respond to an array of stimuli such as electric field, temperature, pH, light, enzyme, or specific chemical signals.^[164]^ Upon optimization, such hybrid nanostructures allow for precise controls over when, where, and how much of the payload is released.

#### 3.1.3. Functionalization with Raman Probes for Multiplex Assay

Inorganic nanoparticles have found use in immunoassay vital to point-of-care testing (POCT) and personalized medicine. In this application, the nanoparticle is conjugated with an antibody and then utilized as a marker or enzyme mimic for detection. As an example, AuNPs have found use in commercial products for POCT, ranging from pregnancy testing to infectious virus detection, diabetes monitoring, cancer screening, and cardiovascular disease assessment.^[175]^ Enabled by their strong and tunable absorption/scattering in the visible region, the colors of AuNPs can be detected by the naked eye. For pregnancy testing, AuNPs are conjugated with antibodies for hCG, a hormone excreted during early pregnancy,^[176]^ to create a red test line for the indication of a positive result. Similarly, during the COVID-19 pandemic, molecular diagnostic kits based on antibody-conjugated AuNPs were developed to enable rapid and cost-effective screening of infected individuals.^[177]^ In one design, AuNPs are conjugated with antisense oligonucleotides (ASOs) to target the N-gene of SARS-CoV-2, the virus responsible for the pandemic. In the presence of SARS-CoV-2, the ASO-capped AuNPs are triggered to agglomerate, giving rise to a discernible color change from violet to blue.

Although colorimetric detection is by far most commonly used in commercial products for immunoassay, the broad (ca. 50 nm) width of an electronic or plasmonic absorption peak makes it difficult to achieve multiplexing for the simultaneous detection of different analytes in a single test.^[178]^ In contrast, the vibrational peaks of molecular species are much narrower (ca. 2 nm), making it attractive for multiplex assay. In particular, the Raman scattering peaks from probe molecules can be utilized for both fingerprinting and multiplexing.^[179]^ When organic molecules are immobilized on the surface of Au or Ag nanoparticles to fabricate SERS tags, their Raman scattering cross section can be enhanced by 5–6 orders in magnitude. Since SERS relies on the unique vibrational peaks of probe molecules for fingerprinting, it does not require other labels. Immunoassay based on SERS tags has the potential to achieve minimal spectral overlap, negligible background noise, and multiplex assay.^[180]^

**Figure 11.**
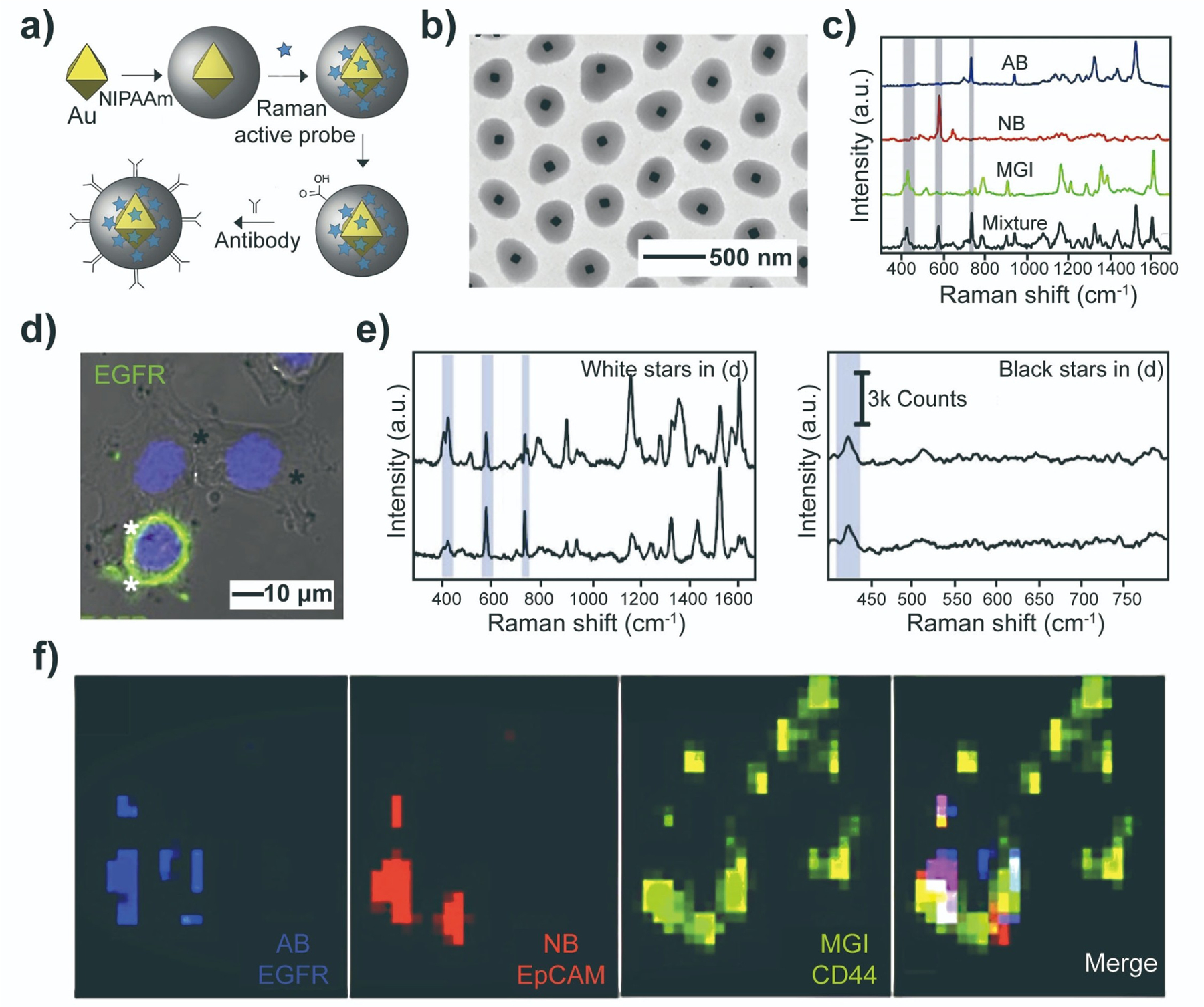
a) Schematic showing the preparation of Au@pNIPAAm SERRS tags. b) TEM image of the tags. c) SERRS spectra of the tags encoded with AB (blue), NB (red), MGI (green), and a mixture of the three. The excitation wavelength was 633 nm. d) Expression of EGFR in cocultured A431 and 3T3 2.2 cells. e) SERRS spectra recorded from the regions marked by white and black stars in (d). f) SERRS mapping at 748 (AB), 592 (NB), and 420 cm^−1^ (MGI) from the same A431 tumor cells. Reproduced with permission from ref. [21]. Copyright 2015 Wiley-VCH.

As an advanced design, surface-enhanced resonance Raman scattering (SERRS) tags offer an even stronger signal enhancement by another 100 folds when the wavelength of the excitation laser matches the absorption peak of the probe molecule.^[21]^ In one study, SERRS tags based on AuNPs were used to simultaneously detect and image three tumor-associated markers: epidermal growth factor receptor (EGFR), epithelial cell adhesion molecule (EpCAM), and homing cell adhesion molecule (CD44), enabling the differentiation of tumor cells from non-tumor cells.^[21]^ The tag was constructed by coating the surface of AuNPs with a pNIPAAm shell encoded with a Raman active probe. The tag was then covalently conjugated with a tumor-targeting antibody (Figure 11a,b). When encoded with a distinct Raman probe such as astra blue (AB), Nile blue (NB), or malachite green isothiocyanate (MGI), the SERRS tags exhibited unique absorption signatures and characteristic Raman peaks at 748, 592, and 420 cm^−1^, respectively (Figure 11c). As such, the three probes can be simultaneously detected and resolved. To evaluate their multiplexing capability for the simultaneous detection of three tumor-associated markers, the AB, NB, and MGI-labeled SERRS tags were functionalized with specific antibodies to target EGFR, EpCAM, and CD44, respectively, and cocultured with A431 tumor (EGFR+/EpCAM+/CD44+) and 3T3 2.2 non-tumor (EGFR-/EpCAM-/CD44+) cells. Confocal microscopy analysis was conducted to differentiate A431 from 3T3 2.2 cells (Figure 11d) so that the areas marked with white and black stars were chosen to perform the multiplexed SERRS analysis. In the case of A431 cells, the SERRS spectra exhibited the characteristic Raman peaks of AB, NB, and MGI (Figure 11e). This implies the simultaneous presence of all three SEERS tags along with their tumor-associated markers. In contrast, only the characteristic Raman peak of MGI was observed in the area marked by a black star in Figure 11d, suggesting that these cells expressed CD44 exclusively to support their assignment as 3T3 2.2 cells. From the spatial distributions of Raman shifts at 748, 592, and 420 cm^−1^ (Figure 11f), it was clear that the anti-EGFR (AB) and anti-EpCAM (NB) SERRS tags were confined to A431 cells, whereas the anti-CD44 (MGI) SERRS tags were observed on both A431 and 3T3 2.2 cells. Altogether, the SERRS tags could differentiate A431 tumor cells from 3T3 2.2 non-tumor cells at a single excitation wavelength.

Due to the strong enhancement in resonance Raman scattering, SERRS tags are well-suited for stable and sensitive multiplex assay and thus cellular heterogeneity profiling. Specifically, SERRS tags equipped with multiplexing capability can reveal intricate subcellular information at high levels of complexity and facilitate spatial profiling. In one study, a library of SERRS tags with 26-plex distinct Raman probes was developed for successful multiplexing and imaging both in vitro and in vivo.^[181]^ Specifically, the SERRS tag was constructed from 60-nm AuNPs labeled with one of the 26-plex probes and further functionalized with a targeting ligand. The fingerprint of each tag can be distinguished from each other in the library to enable multiplexing. The authors demonstrated the ability to target biomarkers and acquired an insight into the spatial relationships among various cell types. Moreover, the tags can be incorporated into chips and functionalized with tumor antibodies to capture and detect cancer cells, enabling multiplexing detection in a single serum droplet of ca. 2 mL with high sensitivity and molecular specificity.^[182]^ Given their multiplexing capability and diverse platforms, SERS tags are expected to play an increasingly important role in shaping the emerging field of personalized medicine.

#### 3.1.4. Fabrication of Nanomotors

Nanomotors refer to nanoscale devices that can convert other forms of energy into mechanical forces for autonomous motions.^[183]^ Benefiting from their autonomous propulsion capability and miniatured size, nanomotors can access regions in the human body conventionally inaccessible and hold promise in a variety of biomedical applications, including diagnostics,^[184]^ drug delivery,^[185]^ and microsurgery.^[186]^ To achieve these goals, the nanomotor must overcome the disturbance from Brownian motion, as well as the environmental drag, to efficiently maneuver toward the target. Externally applied physical fields, such as optical,^[20,76,185]^ acoustic,^[187]^ magnetic,^[28–30]^ and electric,^[75]^ trigger and maneuver the movements of nanomotors through either the directly applied forces or generation of local vector fields that control the flow. Nanomotors also gain kinetic energy by interacting with the surrounding environment by either catalytically reacting with fuel chemicals in the medium^[188]^ or binding to specific signal molecules to initiate self-diffusiophoresis,^[184]^ self-electrophoresis,^[189]^ or bubble propulsion.^[29,190]^ Biohybrid nanomotors have also been proposed, where the intrinsic chemotactic activity of living organisms is harnessed to propel and direct the translocations of nanostructures.^[191]^ Here we focus on nanomotors that do not involve a component directly harvested from biological sources.

Asymmetry is essential for the directional propulsion of most nanomotors. Material hybridization offers a natural route to asymmetry. Typically, the nanomotor adopts an anisotropic structure, with one end serving as the propelling component. Inorganic and organic materials with unique energy conversion capabilities, such as Au,^[20]^ TiO_2_,^[36]^ Fe_3_O_4_,^[28–30]^ Ni,^[68,187,192]^ and perfluorocarbon,^[193]^ have all been used to drive the movement of nanomotors upon the application of an external stimulation. In one study, nanomotors were fabricated by growing Ni nanorods on the PS beads in a closely packed array using the oblique angle deposition method.^[68]^ Under a rotating magnetic field, the mechanical movement of the Ni nanorods enhanced the convective transport of tissue plasminogen activator to the blood clots for the potential treatment of hemorrhage stroke.

Another major category of nanomotors involves catalytic driving or self-propelling. To this end, Pt, which can catalytically decompose H_2_O_2_ into O_2_ and H_2_O, is most commonly used as the propelling element.^[29,190,194]^ A Pt-based nanomotor was fabricated by entrapping Pt nanoparticles in the nanocavities of a bowl-shaped polymer stomatocyte.^[190a]^ With Pt serving as the catalyst, a directional thrust was generated by the rapid production and discharge of O_2_ through the stomatocyte opening. The same design was also extended to other combinations of materials, including MnO_2_ nanoparticles in PEG-*b*-poly(_D,L_-lactide) (PEG-PDLLA) stomatocytes.^[37]^ In an H_2_O_2_-rich environment, the nanomotors showed higher infiltration into cancer cells relative to control particles, demonstrating their potential in targeted delivery. However, the application of these nanomotors is limited due to their reliance on a toxic fuel such as H_2_O_2_ for self-propulsion. To resolve this problem, nanomotors powered by biocatalytic reactions have been developed. For example, in 2015, nanomotors were fabricated from MSNPs, with one hemisphere conjugated with catalase, urease, and glucose oxidase (GOx), respectively.^[35]^ Asymmetric biocatalytic reactions taking place on one side of the nanomotor generated a gradient of chemicals in its ambient solution, resulting in a chemophoretic action. Since the fuels for the latter two enzymes are abundant in natural biological environments, and the hollow space of MSNPs is available for the loading of therapeutic agents, this hybrid nanomotor is promising for biomedical applications. Since this work, a plethora of bio-benign fuel materials, including ATP, collagen, and triglyceride, have been explored and many other nanomotors powered by enzymatic reactions have been reported.^[195]^

In some nanomotor designs, the non-propelling components adopt more functional roles than simply serving as a template for fabrication or structural support. In some cases, they help steer the nanomotors.^[36,193]^ For example, a thermophoretic nanomotor was fabricated with a matchstick structure composed of a black TiO_2_ head and a polymer-coated SiO_2_ nanorod tail.^[36]^ Upon NIR exposure, the TiO_2_ head generates a local thermal gradient, initiating a movement with a tunable magnitude and direction determined by the type of polymer grafted to the tail end.

**Figure 12.**
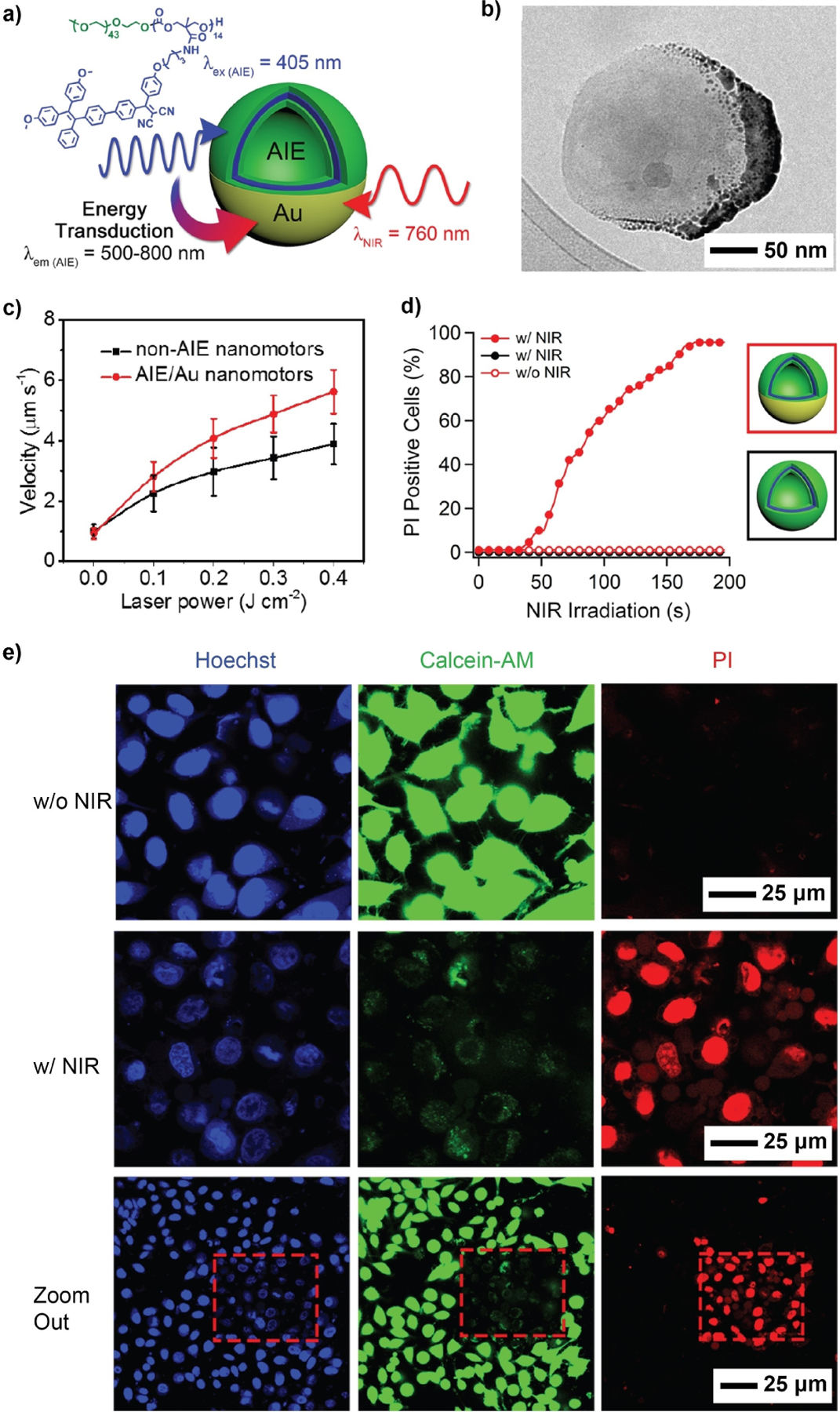
a) Schematic of the AIE/Au hybrid nanomotor, highlighting the synergistic effect between the two components for enhanced motility. b) Cryo-TEM image of an AIE polymersome with a hemispherical Au shell (dark patch). c) Comparison between the velocities of non-AIE nanomotors and AIE/Au nanomotors under different intensities of TP-NIR laser. d) The enhanced capability of AIE/Au nanomotor in triggering cancer cell apoptosis, as demonstrated by the higher percentage of HeLa cells with disrupted cell membrane permeable to PI dye. Data for AIE/Au hybrid nanomotor (red) and AIE polymersomes (black), with and without irradiation, is plotted against the total TP-NIR laser irradiation time. e) Confocal images showing highly localized cell apoptosis caused by nanomotors with or without TP-NIR irradiation. Cell nuclei, live cells, and apoptotic cells were stained by Hoechst, calcein-AM, and PI to give blue, green, and red fluorescence, respectively. Reproduced with permission from ref. [76]. Copyright 2021 Springer Nature.

To endow the nanomotors with enhanced therapeutic effects, many of them were equipped with organic components for the autonomous transportation of bioactive compounds. In general, this could be achieved through two approaches: *i*) conjugating a bioreceptor such as an antibody^[187]^ or DNA probe^[196]^ to the propelling element to capture specific biological elements and *ii*) equipping the nanomotor with a compartment, such as mesoporous SiO_2_,^[197]^ hydrogel,^[194b]^ or polymersome,^[185]^ for housing the therapeutic agent. To take this further, researchers have proposed the use of nanomotors for combination cancer therapy, as exemplified by photoactivated nanomotors with an aggregation-induced emission-genic (AIEgenic) polymersome core and an asymmetrically coated plasmonic Au shell (Figure 12a).^[76]^ AIEgens are a class of compounds that are weakly emissive or non-emissive in a dispersed state but display strong fluorescence upon aggregation. Leveraging on this property, AIEgens have been explored in a range of biomedical applications, particularly, image-guided photo-dynamic therapy.^[198]^ In one design, amphiphilic PEG containing two AIE moieties–tetraphenylethylene and dicyanovinyl moieties–was synthesized and triggered to self-assemble into stable polymersomes using a dropwise solvent switching method. The second functional component, a hemispherical Au nanoshell, was then created by sputter coating. Figure 12b shows a TEM image of the nanomotor. Directional movements of the AIE/Au hybrid system were driven by the thermal gradient arising from the asymmetric local plasmonic heating at the Au-coated side under two-photon near-infrared (TP-NIR) irradiation. Notably, the velocity of the hybrid system was 45 % greater than that of the Au-coated polymersomes without AIE moieties, indicating a synergistic effect between the inorganic and organic components (Figure 12c). Further investigation revealed that the polymeric AIE framework was excited, transducing its radiant energy to the Au shell and thus bolstering the thermophoresis of the hybrid nanomotor. Following the validation of their motility, the authors applied these nanomotors to photodynamic therapy. As indicated by the high levels of propidium iodide (PI) sequestration (Figure 12d,e), the HeLa cancer cells treated with AIE/Au hybrid nanomotors underwent catastrophic membrane disruption upon TP-NIR irradiation for 200 s. The subsequent cell apoptosis was highly localized to the area exposed to the laser, with the viability of the surrounding cells under the influence of “dormant” nanomotors unimpaired. Altogether, this hybrid design highlighted the functional synergy between its two components in both augmenting motility and enhancing therapeutic efficiency.

A major challenge faced by nanomotors in biomedical applications, particularly, for in vivo ones, is their real-time tracking. To resolve this issue, some researchers took advantage of the intrinsic properties of the materials of the nanomotors and utilized MRI, ultrasonic imaging, CT, and PA imaging to track their locations.^[199]^ Others took a more active approach of purposely incorporating fluorescent elements into the nanomotors, enabling fluorescence tracking with high spatiotemporal resolution. To this end, organic chromophores, QDs, and MOFs with fluorescent moieties are either grafted to the surface of the nanomotors or impregnated/doped into their structure to grant them fluorescent trackability.^[200]^

As illustrated in all the aforementioned examples, the concept of material hybridization is key to nanomotor development. Not only is it pivotal to achieving the basic motility of nanomotors, but more importantly, it empowers these nanomachines with great potential such as enhanced therapeutic effects for biomedical applications.

#### 3.1.5. Contrast Agents for Multi-Modal Imaging

Hybrid nanomaterials with multifunctionality tailored through diverse modifications are driving innovations in the field of molecular imaging through the achievement of multi-modality. These innovations have major implications not only for the field of disease diagnosis but also for the comprehensive monitoring of intricate biological processes in living organisms.^[201]^

With the aid of contrast agents or probes, MRI stands out as one of the most potent tools for monitoring the state of organs like the brain and heart, as well as for assessing the type and stage of tumors. This can be attributed to its remarkable high-resolution capability and unparalleled penetration depth.^[202]^ Nonetheless, certain limitations persist. For instance, low molecular weight Gd chelates, one of the conventional MRI contrast agents, tend to accumulate in ECM, potentially disrupting the blood–brain barrier.^[203]^ Additionally, the rapid diffusion of Gd chelates results in imprecise lesion localization, necessitating the repetitive administration of high doses for effective contrast enhancement.^[204]^ It remains a challenge to address these adverse effects associated with MRI. In contrast, intraoperative optical imaging, relying on either intrinsic tissue optical properties or exogenous contrast agents, offers the advantages of being rapid, low-cost, and highly sensitive. However, optical methods are limited by spatial resolution and penetration depth. Recently, PA imaging has emerged as an advanced technique that overcomes the challenges in optical imaging.^[205]^ During PA imaging, the tissue undergoes heat absorption and subsequent thermoelastic expansion for the generation of acoustic waves. The acoustic signals are collected and constructed to map the optical absorption information of the tissue.^[206]^

**Figure 13.**
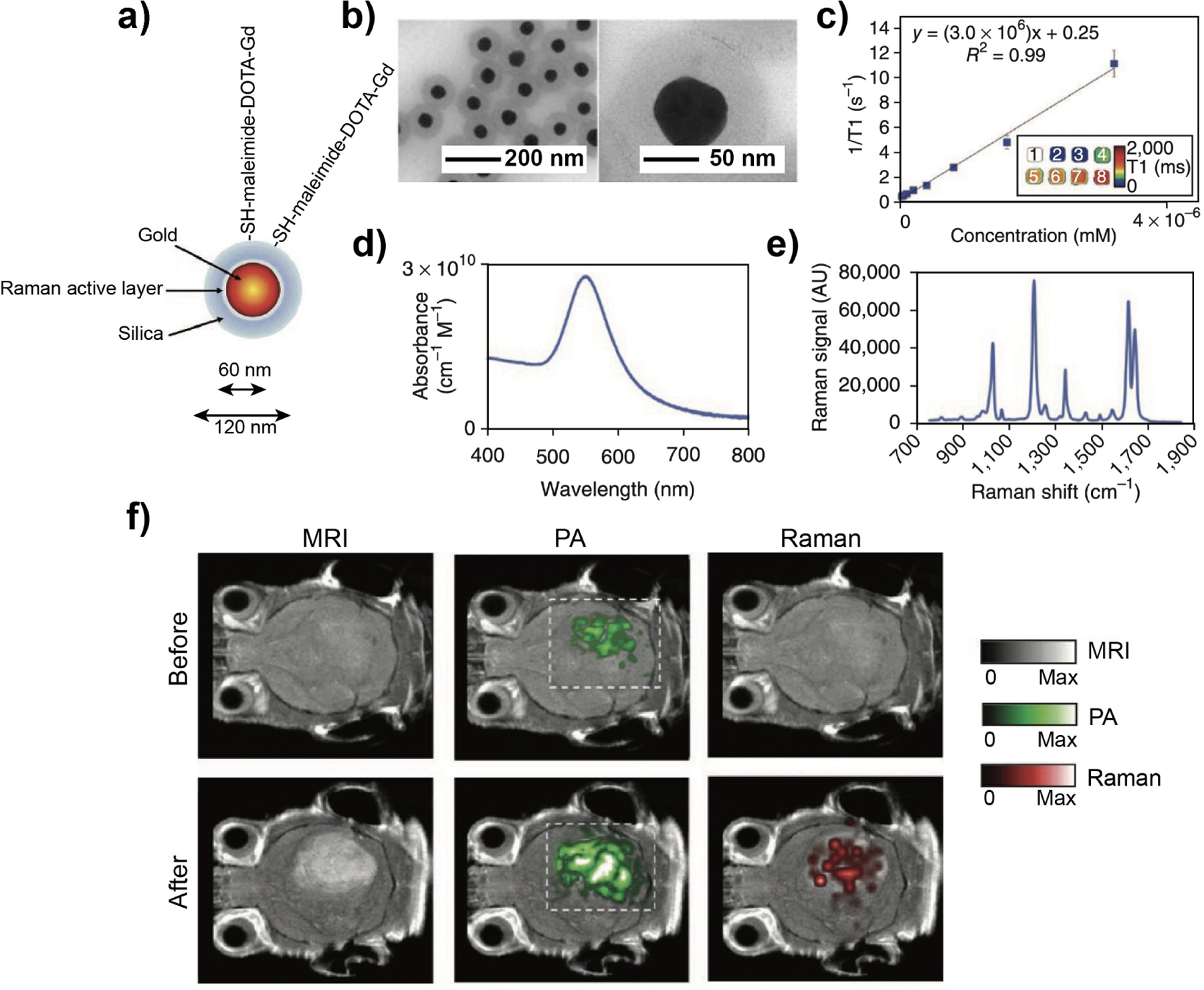
a) A simplified diagram of the MPR contrast agent. b) TEM images of the nanoparticles. c) Particle relaxivity generated from T1 mapping of probe dilutions in an MRI phantom. d) Absorption spectrum of the nanoparticles. e) Raman spectrum of the nanoparticles. f) Two-dimensional axial MRI, PA, and Raman images. Reproduced with permission from ref. [43]. Copyright 2015 Springer Nature.

Given that each imaging modality possesses distinct strengths and weaknesses, an integrated platform becomes imperative to enhance diagnostic accuracy. Such an integration allows for the acquisition of complementary information from different imaging modalities, contributing to a comprehensive assessment. To this end, there is an urgent need to fabricate multifunctional nanoparticles that support a variety of properties, including magnetic, electronic, and optical, among others.^[207]^ The multifunctionality calls for hybridization of different organic and inorganic components. One study developed a multifunctional nanoparticle, referred to as MRI-PA-Raman (MPR) contrast agent (Figure 13a), which embraces the imaging capabilities of MRI, PA, and Raman.^[43]^ This hybrid system was effective in accurately defining the boundaries of brain tumors in living mice, offering a valuable tool for both preoperative and intraoperative tumor margin delineation. The MPR agent consisted of 60-nm AuNPs covered by a 30-nm SiO_2_ shell containing a Raman probe and further modified by 1,4,7,10-tetraazacyclododecane1,4,7,10-tetraacetic acid (DOTA)-Gd^3+^ (Figure 13b). The nanoparticles exhibited a high T1 relaxivity of 3.0×10^6^ mM^−1^ s^−1^ (Figure 13c), serving as a contrast agent for T1 MRI in vivo. The nanoparticles showed an optical absorption peak at 540 nm (Figure 13d), making them a contrast agent for PA imaging. Moreover, the nanoparticles possessed a unique Raman signature for SERS imaging (Figure 13e). To further assess the multimodal imaging performance of the hybrid nanoparticles, the authors administered them to tumor-bearing mice. The after-injection images demonstrated visualization of the tumor across all three modalities, even when acquired through highly attenuating tissues such as intact skin and skulls (Figure 13f). Significantly, both the PA and SERS images were co-registered with the MRI image, illustrating robust colocalization and registration among the three modalities. The integration of MRI, PA, and Raman imaging promises to improve the accuracy of brain tumor resection by synergistically harnessing the strengths inherent to each of these modalities.

In other demonstrations, SPIO nanoparticles were coupled with optical agents or radioactive isotopes and explored for multi-modal imaging, encompassing combinations such as MRI/optical or MRI/nuclear imaging.^[208]^ These multifunctional contrast agents offer a precise and clear demarcation of tumor-associated vessels and the tumor microenvironment. Hybridized multi-functional nanoparticles not only serve as versatile multi-modal contrast agents but also play a pivotal role in advancing image-guided therapies through their adept utilization of distinct physical and chemical properties. Taken together, hybrid nanomaterials offer a natural vehicle to integrate the diverse contrast agents into one compact system for easy administration. The advancement of hybrid nanomaterials in the realm of multimodal imaging holds the promise of spearheading the next-generation disease diagnosis and therapy, fostering sustainable progress in healthcare.

#### 3.1.6. Theranostic Agents for Image-Guided Therapy

Theranostics represents a medical approach that integrates diagnostics (e.g., imaging or sensing) with therapy to treat disease more effectively than the conventional procedure.^[209]^ It has the potential to personalize the treatment for an individual patient, enhancing comprehensive treatment effectiveness while minimizing side effects. To this end, hybrid nanomaterials offer incredible capability and flexibility due to the rich variety of possible combinations of components. For example, one of the components can serve as a probe or contrast agent for imaging while the other component can exhibit a therapeutic effect or contain a therapeutic agent. Furthermore, coating with proper surface ligands could equip them with prolonged circulation, improved biodistribution, and enhanced targeting capability.^[210]^ In some cases, by combining different types of nanomaterials, the hybrid system can exhibit synergistic properties not present in individual components, streamlining the theranostics process.^[211]^

**Figure 14.**
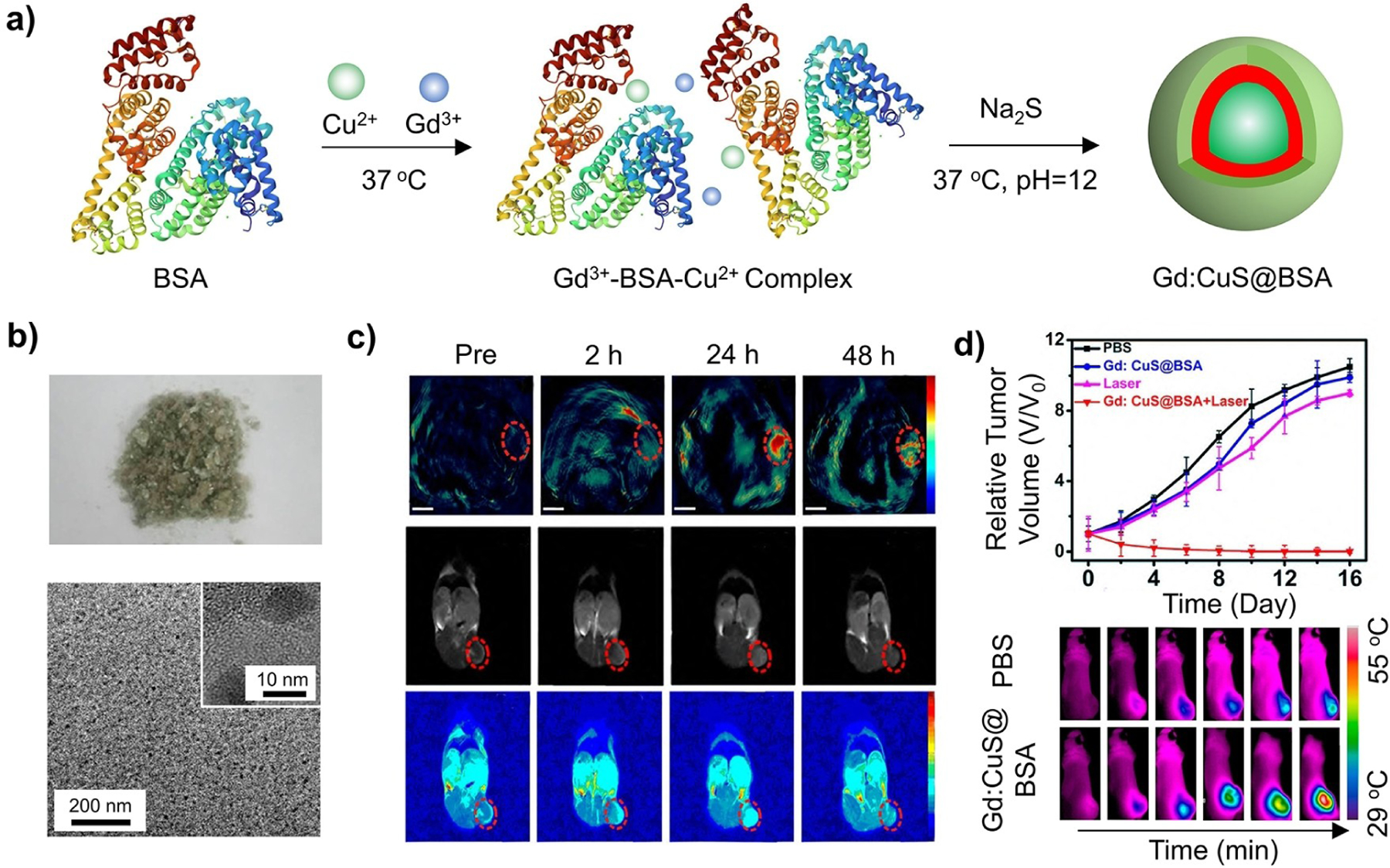
a) Schematic of the synthetic approach. b) Digital photo, TEM, and high-resolution TEM (inset) images of the lyophilized Gd:CuS@BSA nanoparticles. c) Time-elapsed in vivo PA/MR dual-modal imaging in tumor-bearing mice before and after intravenous injection of 150-μL of Gd: CuS@BSA at 2, 24, and 48 h post-injection. Scale bar = 3 mm. d) Relative tumor growth curves during photothermal therapy. The bottom panel shows thermal images of the tumor-bearing mice before and after intravenous injection with PBS or Gd:CuS@BSA, followed by irradiation with a 980-nm diode laser for 5 min. Reproduced with permission from ref. [44]. Copyright 2016 American Chemical Society.

In general, the hybridization of inorganic and organic components allows enhanced bioimaging capabilities and disease-specific targeting ability for precision treatment. Among them, combination therapy, a procedure that synergistically combines multiple therapeutic modalities to achieve the optimal outcome, captured significant attention. Using bovine serum albumin (BSA) as a template, one study developed a hybrid system comprised of Gd-doped CuS nanoparticles (Figure 14a,b).^[44]^ The high binding affinity of BSA toward transition metal ions plays a crucial role in effectively recruiting Cu^2+^ and Gd^3+^ species, circumventing the requirements for high temperatures (> 90 °C) and toxic ligands during the synthesis. Owing to its natural occurrence, the BSA layer on the surface not only enhances the stability of the resultant nanoparticles and reduces agglomeration, but also improves biocompatibility, obviating the need for additional surface modification to achieve efficient circulation and targeting efficacy. The CuS component shows strong optical absorption in the NIR region, leading to easy visualization by PA imaging and effective photothermal treatment, while the Gd^3+^ species with unpaired electrons are known for their ability to enhance the tissue MRI visibility and imaging contrast. To evaluate the dual-modal imaging capability, mice bearing SK-OV-3 tumors were intravenously administrated with 150 μL of the Gd:CuS@BSA nanoparticles in PBS. The injected hybrid nanoparticles accumulate in the tumor tissue due to the enhanced permeability and retention effect. The average PA intensity increased continuously until 24 h, accounting for a 9-fold enhancement relative to the signal before injection. Similarly, the MRI enhancement at the tumor site kept increasing until 24 h, and the intensity of the T1 signal (known as longitudinal relaxivity) increased by 1.4-, 2.25-, and 1.5-fold at 2, 24, and 48 h, respectively (Figure 14c). In addition, the Gd:CuS@BSA nanoparticles showed exceptional photothermal therapy capabilities. The tumors containing Gd:CuS@BSA exhibited remarkable regression two days after NIR laser treatment and were eradicated by day 6. In contrast, the tumors in the control groups, treated with PBS, Gd:CuS@BSA only, or NIR laser only, showed rapid growth (Figure 14d). Altogether, the findings demonstrate that this hybrid system exhibits considerable potential in providing imaging guidance and effective tumor ablation capabilities with high spatial resolution and deep tissue penetration.

Overall, there is a strong desire to develop hybrid systems for more efficient, intelligent, and safer multiplexable theranostics.^[212]^ Such systems can integrate various imaging and therapeutic modalities, such as CT, ultrasound, PET, single-photon emission computed tomography, and PA imaging, along with radiotherapy, immunotherapy, and chemotherapy, among others. To achieve diverse combinations, hybridized theranostic systems must be designed with carefully selected targeting ligands, imaging probes, and therapeutic agents to ensure synergy among the components without compromising or interfering with each other.

### 3.2. Nanocomposites

We choose a set of examples to illustrate how inorganic nanostructures are embedded in a variety of organic matrices to enhance and/or enrich their biomedical applications in the context of wound management, stretchable bioelectronics, actuating, and tissue regeneration.

#### 3.2.1. Dressing Materials for Wound Management

Serving as the foremost natural protective barrier, skin diligently upholds the body’s homeostatic balance by responding to environmental changes.^[213]^ When experiencing injuries like burns, trauma, surgical incisions, or exposure to hazardous compounds, the structural integrity of the skin will be compromised, resulting in wound formation. Severe conditions, such as inflammatory infection and vascular or neurological dysfunction, would cause the wound to deteriorate into chronic ones, making it challenging for effective wound management.^[214]^ Although widely employed in clinical practice, antibiotics can exert selective survival pressure on bacteria, leading to the development of drug resistance. It has been an ongoing endeavor for several decades to explore nanocomposites as dressing materials for wound management.^[215]^ The nanocomposites can be engineered with distinct qualities, including antioxidative activity,^[216]^ antibacterial capability, and pro-angiogenic effect,^[217]^ all of which can work collectively to regulate the microenvironment of the wound and thereby facilitate effective wound healing.

Nanocomposites have emerged as a promising choice for wound management due to their remarkable flexibility, water-absorbing and retaining capacity, and excellent biocompatibility.^[218]^ As for the matrices, polymers,^[219]^ extracellular vesicle-based matrices,^[220]^ and hydrogels all stand out as excellent choices. In particular, hydrogels, which contain a 3D network of polymer chains, are known for their high water content, exceptional flexibility, and malleability. The unique 3D structure of hydrogels, when combined with bioactive functional nanomaterials, opens up new opportunities to augment wound healing.^[221]^

Nanomaterials have also advanced the realm of wound management by offering highly effective antimicrobial treatments through methods such as chemo, photodynamic, photothermal, and immune therapies.^[222]^ Specifically, in response to noninvasive NIR irradiation, nanomaterials capable of light-to-heat conversion can produce localized hyperthermia for photothermal therapy, while simultaneously generating ROS for photodynamic therapy.^[223]^ The dual mechanisms can work together to effectively cause structural disruption in bacteria. To further tackle the challenges related to insufficient specificity and limited penetration depth of optical treatment, researchers have harnessed the power of highly toxic hydroxyl radical (·OH) derived from a Fenton-like reaction during chemodynamic therapy. The nanomaterials synergistically combine photo-thermal, photodynamic, and chemo methods, greatly enhancing the overall efficacy.^[224]^

**Figure 15.**
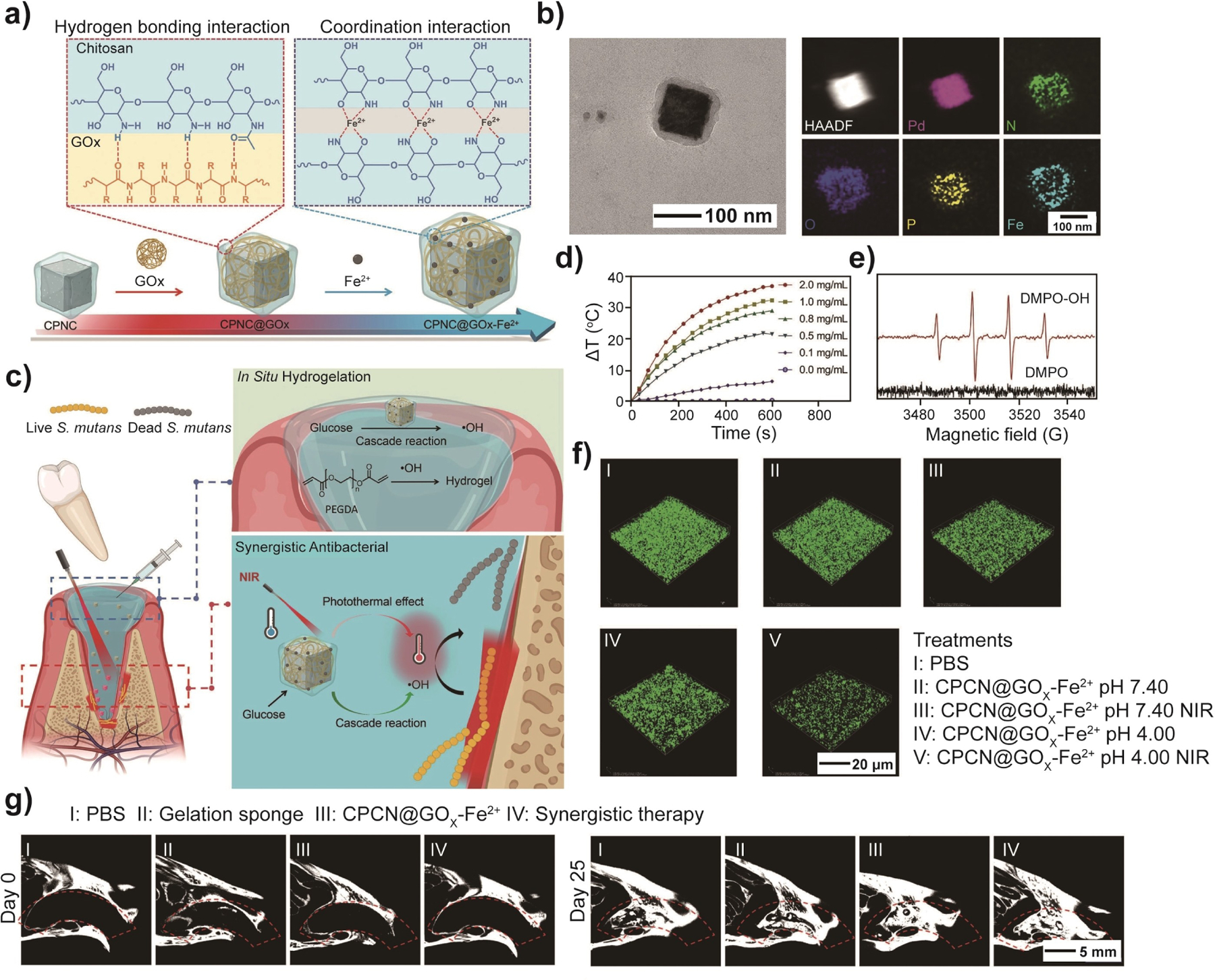
a) Schematic showing the synthesis of a photothermal cascade nanoreactor denoted CPNC@GOx-Fe^2+^. b) TEM image and EDX mapping of a CPNC@GOx-Fe^2+^. c) Schematic illustrating the adoption of CPNC@GOx-Fe^2+^ to augment tooth-extraction wound healing. d) Plots showing the photothermal activity of CPNC@GOx-Fe^2+^ with different CPNC contents under NIR irradiation (808 nm, 1 W cm^−2^). e) Electron paramagnetic resonance spectra of ·OH produced by the cascade reaction. f) Green fluorescent nucleic acid staining images of *S. mutans* biofilms after various treatments. g) Mirco-CT images of the sagittal plane of tooth extraction wound in rats and grayscale statistics at tooth extraction wound. Reproduced with permission from ref. [25]. Copyright 2023 Wiley-VCH.

The synergy between the nanoscale component and organic matrix in a nanocomposite is of great importance for comprehensive wound management. One study reported a nanocomposite self-assembled from GOx, Fe^2+^ ions, and chitosan-modified Pd nanocubes (CPNC), and further protected by a poly(ethylene glycol) diacrylate (PEGDA) hydrogel. This nanocomposite facilitated potent antibacterial therapy and accelerated the wound-healing process following tooth extraction (Figure 15a).^[25]^ In the design, CPNC served as a photothermal agent. Through a combination of hydrogen bonding and coordination interactions, GOx and Fe^2+^ were immobilized on the chitosan surface (Figure 15b), enhancing its resistance to enzymatic degradation while improving its overall thermostability. In addition to its role in catalyzing the oxidation of glucose for the production of ·OH radicals, CPNC@GOx-Fe^2+^ also exhibited potent photothermal effects on bacteria when exposed to a NIR laser. The as-generated ·OH could act as an initiator for the in situ formation of PEGDA. The polymerization led to the formation of a PEGDA hydrogel for the protection of the open wound (Figure 15c). During continuous NIR irradiation, the CPNC@GOx-Fe^2+^ suspension showed a gradual increase in temperature, as evidenced by the concentration-dependent trend (Figure 15d). The electron paramagnetic resonance spectra in Figure 15e confirmed the generation of ·OH, which served as the foundation for chemodynamic antibacterial therapy. A tooth-extraction wound model was engineered to explore the synergistic anti-bacteria and wound healing capability of CPNC@GOx-Fe^2+^ in vivo. The synergistic group (CPNC@GOx-Fe^2+^-hydrogel under irradiation) exhibited a lower relative abundance of bacteria relative to the other groups (Figure 15f), indicating the effective antibacterial capability of this nanocomposite in wound management. Micro-CT images revealed noticeable healing in the synergistic group for tooth-extraction wounds (Figure 15g). Collectively, these results validate the efficacy of this hybrid system for managing tooth-extraction wounds through a synergistic approach that combines photothermal and chemodynamic therapies.

In another example, a similar nanocomposite was developed by combining CaO_2_ particles with indocyanine green, lauric acid, and MnO_2_. This composite was then encapsulated in a hydrogel comprised of PDA and hyaluronic acid.^[38]^ Upon exposure to a NIR laser, lauric acid was induced to swell, exposing CaO_2_ to water for the production of O_2_ under the catalysis of MnO_2_. Owing to its responsiveness to NIR light, indocyanine green facilitated the generation of singlet oxygen, effectively suppressing inflammation outbreaks for wound management. Similarly, a hollow Cu_2-X_S nano-homojunction platform that supports multimode dynamic therapies also showed promise as a candidate for both wound healing and cancer therapy.^[225]^ It is expected that further optimization through the integration of precisely engineered inorganic nanomaterials, biomimetic matrices, and advanced fabrication processes will advance wound management.

#### 3.2.2. Conductive Materials for Stretchable Bioelectronics

Stretchable bioelectronics, also known as elastic bioelectronics or elastic circuits, refers to electronic circuits deposited on or embedded in elastic substrates such as silicones or polyurethanes to accommodate large strains without failure.^[226]^ In general, the term “stretchable” encompasses both flexibility and stretchability, like in the case of an elastic polymer film with relatively low (< 10 MPa) Young’s modulus.^[227]^ Due to their flexibility, such bioelectronic circuits can be tightly attached to soft curved organs (e.g., heart, brain, and skin), achieving high-quality interfaces between tissues and monitoring devices.^[228]^ The design of stretchable bioelectronics calls for rational hybridization of inorganic conductive nanofillers with organic soft matrices.^[229]^ In this aspect, the organic component (e.g., conductive polymer or elastomer) offers stretchability and biocompatibility to interact with a biological organ or tissue, enabling the integrated devices to stretch and bend without dysfunction. The inorganic nanofiller (e.g., metal nanostructures or carbon materials), on the other hand, provides the desired conductivity. The hybrid system can be tailored to fit various wearable and/or implantable biomedical occasions for the recording of a range of chemical, mechanical, optical, and electrical signals.^[230]^

The most commonly reported stretchable bioelectronics involve embedding micro- or nanostructured inorganic interconnects in silicone-based implants. Such devices are designed to mimic the mechanical properties of soft human tissues, enabling the diagnosis and treatment of various diseases. One study reported the fabrication of stretchable bioelectronics to mimic dura mater, the protective membrane of the brain and spinal cord, as a long-term multimodal neuroprosthetic.^[26]^ Hybrid soft Pt/silicone electrodes and stretchable Au nano-interconnects are integrated into a flexible silicone substrate for transmitting electrical excitations and transferring electrophysiological signals, respectively. As demonstrated in the rat spinal cord injury model, such a hybrid system enables high-resolution neuronal recording and electrical neuromodulation for more than six weeks.

**Figure 16.**
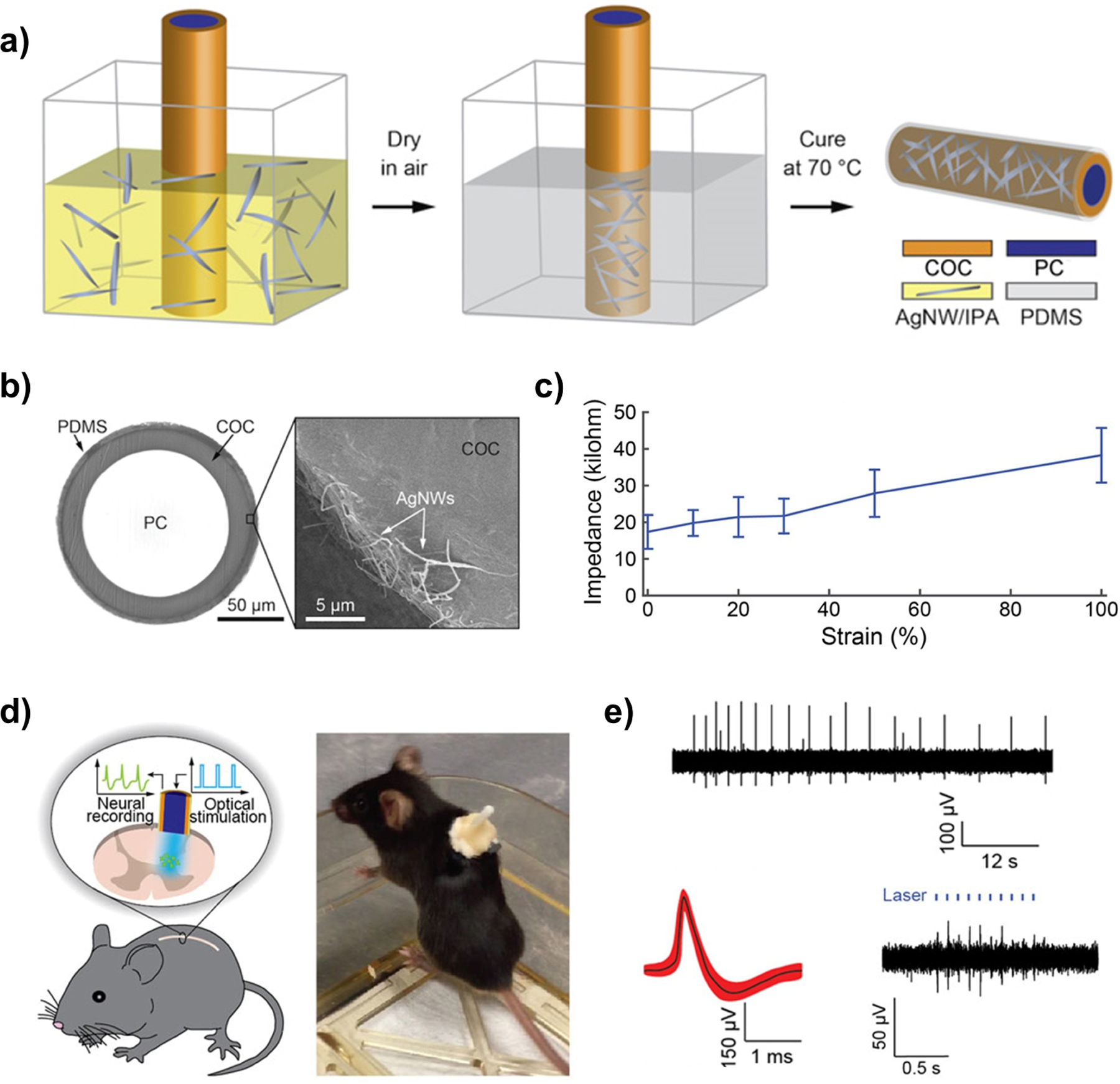
a) Schematic showing the fabrication of a flexible, stretchable, and implantable fiber probe. b) Cross-sectional optical image and corresponding scanning electron microscopic (SEM) image of the probe. c) Correlation between the electrical impedance and stretching strain of the probe. d) Illustration (left) and image (right) depicting the implantation of the fiber probe into the spinal cord of a mouse. e) Recorded spontaneous activity, action potentials, and optically-evoked action potentials measured using the fiber probe implanted in the spinal cord of a wild-type mouse. Reproduced with permission from ref. [24]. Copyright 2017 AAAS.

Another study fabricated a hybrid implantable probe consisting of a conductive mesh of AgNWs coated on the surface of flexible and stretchable polymer fibers to investigate and interrogate neuronal circuits within the spinal cord.^[24]^ As shown in Figure 16a,b, thermal drawing was employed to fabricate fibers comprising a PC core and a COC cladding, followed by dip-coating to deposit a micrometer-thick mesh of AgNWs and finally, encapsulation in a PDMS sheath. In this case, AgNWs were incorporated to create conductive pathways, allowing for the transmission of electrical signals within a flexible and stretchable PC/COC/PDMS matrix. In addition, AgNWs were resilient to stretching deformation and bending, leading to bioelectronic probes capable of maintaining a low impedance of 40 kΩ at strains up to 100 % (Figure 16c). This result underscores the effectiveness of hybrid nanocomposite as a stretchable conductor due to the intrinsic flexibility associated with the mesh of AgNWs (Figure 16c). Because the spinal cord and peripheral nerves only experience strains up to ca. 12 %, the hybrid implantable probes provide arbitrarily scalable and stretchable alternatives to metallic electrodes. After implantation into the spinal cord of a free-moving mouse (Figure 16d), the probe was used to record neural activity, including sensory-evoked potentials, isolated action potentials, and optically-evoked potentials, while enabling optical manipulation of the hindlimb muscles (Figure 16e). Altogether, this hybrid probe could retain a minimal optical transmission loss in the visible spectrum and possess impedance well-suited for extracellular recording even under strains surpassing those observed in mammalian spinal cords. Additional studies revealed no instances of tissue erosion or cytotoxic effects at the interfaces of the fiber implant, demonstrating the viability of such a hybrid probe for stimulating and monitoring electrophysiological activity in the spinal cord.

Despite the advancement, many practical challenges still remain. For example, there still exists a large discrepancy in terms of mechanical stiffness between a major organ such as the brain (1–4 kPa) or heart (10–15 kPa) and the common stretchable matrices, hindering the monolithic conformal integration of the biomedical device with the human body, and even causing various side effects. In addition, many designs are prone to device failure when subjected to significant or prolonged mechanical stress because of their limited self-healing and self-recovery properties. Furthermore, despite ongoing efforts to simultaneously incorporate therapeutic and diagnostic functions into a single system, most stretchable bioelectronics lack spatiotemporal control of drug delivery.^[231]^ While the choices of polymer matrices and inorganic nanomaterials offer unlimited possibilities, comprehensive considerations in the novel use of hybrid nanocomposites and their assemblies, surface/interface, device designs, and even long-term biocompatibility are important for future studies.^[232]^ Advancement in the material hybridization scheme and processing will lead to the development of pliable and stretchable bioelectronics, whose paradigm is poised to expand the horizons of existing healthcare diagnostic and therapeutic technologies.^[233]^

#### 3.2.3. Smart Films for the Capture and Release of Biological Samples

In biomedical applications, specific types of cells, such as circulating tumor cells, immune cells, and stem cells, are often extracted from biological samples like blood, tissues, or organs for subsequent analysis or therapeutic procedures.^[234]^ Smart films constructed from stimuli-responsive inorganic materials, soft polymeric materials, and targeting ligands or antibodies have demonstrated the ability to capture and, at times, manipulate and transport specific cells, while maintaining their integrity and viability.^[235]^ To enhance mobility, soft organic matrices are usually designed with compliance and continuum deformation capabilities. Meanwhile, the embedded inorganic nanomaterials can provide precise feedback controls by leveraging their optical, electrical, and magnetic properties.^[236]^

Several studies involving smart films based on hybrid nanomaterials have demonstrated the ability to perform grasping through actuation or controlled adhesion to the targeted cells.^[237]^ In one study, a photo-responsive smart film was developed to precisely exert mechanical stress on cells. The nanocomposite film contained a temperature-responsive pNIPAAm hydrogel and a hydrogel matrix that was pre-loaded with AuNRs.^[22]^ The AuNRs exhibited strong absorption at wavelengths of 650–900 nm and could release a large amount of heat due to their photothermal effect, forcing the hybrid hydrogel to undergo a reversible volume change above its LCST (ca. 32 °C). Upon illumination by light at the absorption peak, the local temperature quickly rose above the LCST, causing dimensional changes to the composite film. According to an in vitro study, the hybrid platform allowed one to remotely apply uniaxial tension to defined portions of single cells that could be controllably oriented on the substrate at specific locations, with a response time of less than 3 s. When further powered with cell-specific attachment, such hybrid films offer a means to dynamically control cellular attributes like morphology, positioning, and alignment, catering to the unique demands of each application.

**Figure 17.**
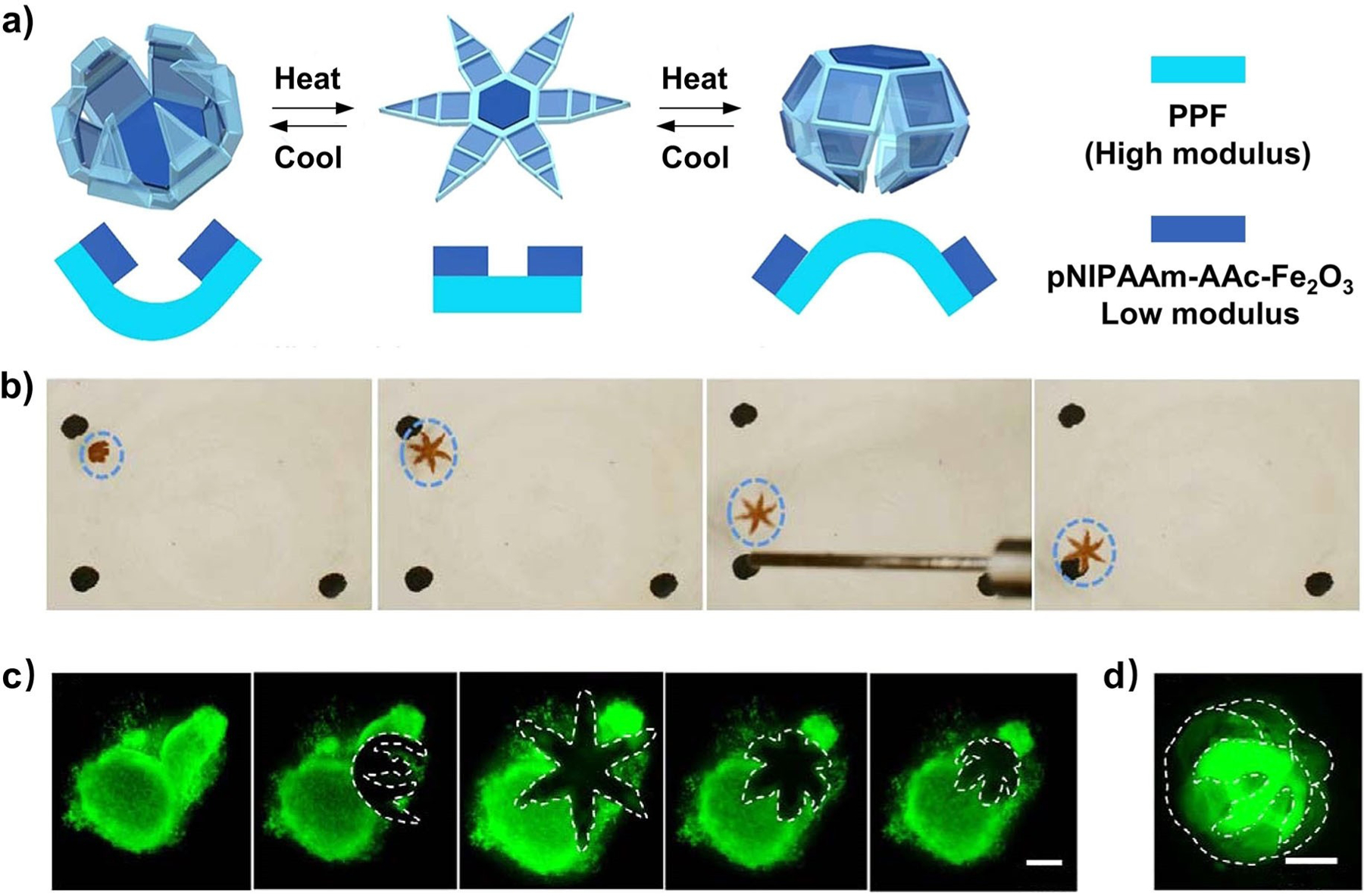
a) Schematic illustrating the reversible self-folding of hybrid smart films comprised of a stiff PPF layer and a magnetic pNIPAAm-AAc-Fe_2_O_3_ layer in response to temperature change. Above the LCST, the pNIPAAm-AAc-Fe_2_O_3_ layer undergoes water exclusion and contraction, forcing the grippers to open and then close. Below LCST, the pNIPAAm-AAc layer absorbs water and swells, causing the grippers to open and then close in the opposite direction. b) Snapshots depicting the magnetic-guided movement of the robot. c) Fluorescence images involving the cell capture and excision from a cluster of live fibroblast cells (scale bar: 1 mm). d) Fluorescence image of a robot with the excised cells in capture (scale bar: 500 μm). Dashed lines are included to assist in visualizing both the grippers and the excised cells. Reproduced with permission from ref. [33]. Copyright 2021 American Chemical Society.

As an energy source for clinically relevant imaging methods, magnetic fields stand out as a stimulus that exhibits good penetration depth and superior safety.^[238]^ A plethora of magnetically-actuated hybrid smart films have been tailored for biomedical applications,^[239]^ including cell capture, transport, and release.^[33]^ Recently, SPIO nanoparticles (5 wt%) were incorporated into a thermo-responsive poly(NIPAAm-*co*-acrylic acid) (pNIPAAm-AAc) hydrogel to obtain magnetic field-responsive microgrippers. To ensure sufficient stability and rigidity for the hybrid robot, the design further combined the pNIPAAm-AAc-Fe_2_O_3_ layer (shear modulus: 162 kPa) with a degradable yet stiff polypropylene fumarate film (PPF, 16 MPa in shear modulus) using photolithography (Figure 17a). As shown in Figure 17b, an external magnet could be used to guide the translocation of the soft microgripper. Significantly, owing to the enhanced rigidity by combining inorganic nanoparticles with solid PPF polymers, this system could effectively grasp and excise cells from a cluster of fibroblast cells (Figure 17c). To illustrate its heating capability, the robot was stored at 4 °C to ensure maximal water absorption by the pNIPAAm-AAc-Fe_2_O_3_ layer that would adopt a full folding conformation. Subsequently, it was placed on a fluorescently labeled fibroblast cluster in warm PBS (37 °C) and allowed to unfold and then close in cold PBS (4 °C), encapsulating the tissue in the grasp. The fluorescent live cells could be visualized through the transparent gripper arms (Figure 17d).

Despite recent progress, the challenge of developing photothermally reprogrammable actuators with precise controls over time and space persists. Researchers need to prioritize the creation of devices that exhibit rapid response times and the capability for multiple programmable actions in response to different external stimuli. Currently, researchers are working on refining existing methods and developing new ones, integrating the hybrid concept and advanced techniques from areas such as microfluidics, nanotechnology, and biophysics.^[240]^ As technology evolves, the ability to precisely capture and release cells will play a pivotal role in advancing various aspects of biology, medicine, and biotechnology, ultimately leading to improved diagnostics, therapies, and insights into cellular behavior.^[241]^

#### 3.2.4. Functionally-Graded Nanocomposites for Tissue Regeneration

Gradient refers to a feature by which a substance or property varies gradually along one or multiple directions. It is prevalent in biological systems, especially in interfacial tissues. In these transition zones, the gradients in composition and/or structure give rise to a gradual change in mechanical properties, together with spatial variations in the concentration of morphogen (i.e., the biochemical substance that directs the lineage of differentiating cells) to induce and maintain a graded transition of cell phenotype.^[242]^ Functionally-graded tissues, exemplified by tendon-to-bone enthesis and dentin-enamel-junction (DEJ), are critical for our daily activities. Disruption of these structures has detrimental effects and requires immediate medical attention.

In traditional tissue engineering, uniform grafts constructed from purely organic or inorganic materials are utilized and surgically implanted to help regenerate the damaged tissue and ultimately restore its functions. However, failure to recapitulate the native gradient usually leads to a buildup of mechanical stress at the interface, resulting in structural breakdown before full regeneration of the original tissue. Moreover, the uniform scaffolds cannot provide adequate guidance to direct the differentiation and eventual functionalization of the stem cells seeded on them or recruited from the surrounding tissues. As a result, it is essential to develop a structure that mimics the natural gradient at the tissue interface to better facilitate the repair or regeneration.

Hybrid nanocomposites have emerged as an attractive alternative for fabricating biomimetic scaffolds. Their popularity stems from their straightforward fabrication process and the ease with which the gradient can be finely adjusted. Altering the composition of the nanocomposite allows for a concurrent adjustment of its physicochemical properties. This capacity enables the design of materials that mimic the characteristics of the native tissues to provide the appropriate biochemical and/or biophysical cues for the cells, leading to the eventual restoration of the damaged tissue.

Stem cell lineage specifications are extremely sensitive to the microenvironments. It was reported that the elasticity of ECM had a major impact on the differentiation of the mesenchymal stem cells (MSCs) seeded on it, dictating the phenotype these cells would commit to.^[243]^ Many studies have shown that nanocomposites with tunable mechanical, electrical, and biochemical properties could be used to manipulate stem cell differentiation. For instance, nanocomposites with carbon nanomaterials such as graphene and CNTs could effectively promote osteogenesis,^[244]^ myogenesis,^[245]^ as well as periodontal^[246]^ and neuronal^[247]^ stem cell differentiation.^[248]^ In one study, GO/PCL nanocomposite was used to guide the differentiation of neural stem cells (NSCs) into oligodendrocytes.^[47]^ Increasing GO concentration led to enhanced expression of key neural markers, indicating the formation of more mature oligodendrocytes. Nanocomposites involving metal nanostructures have also been explored to regulate stem cell differentiation. In particular, nanocomposites comprising AuNPs in BSA-PVA matrix^[53]^ and GelMA hydrogel^[54]^ were used to trigger cardiomyogenic and osteogenic differentiation, respectively, in an Au content-dependent manner. Along a similar direction, our group developed a nanofiber scaffold with a mineral gradient to induce graded osteogenesis of adipose-derived mesenchymal stem cells (ASCs).^[52]^ Specifically, a mat of uniaxially aligned PLGA electrospun nanofibers was coated with a mineral gradient along the axial direction. Osteogenic differentiation of ASCs was positively correlated with the gradient in mineral content, mimicking that in native enthesis.

**Figure 18.**
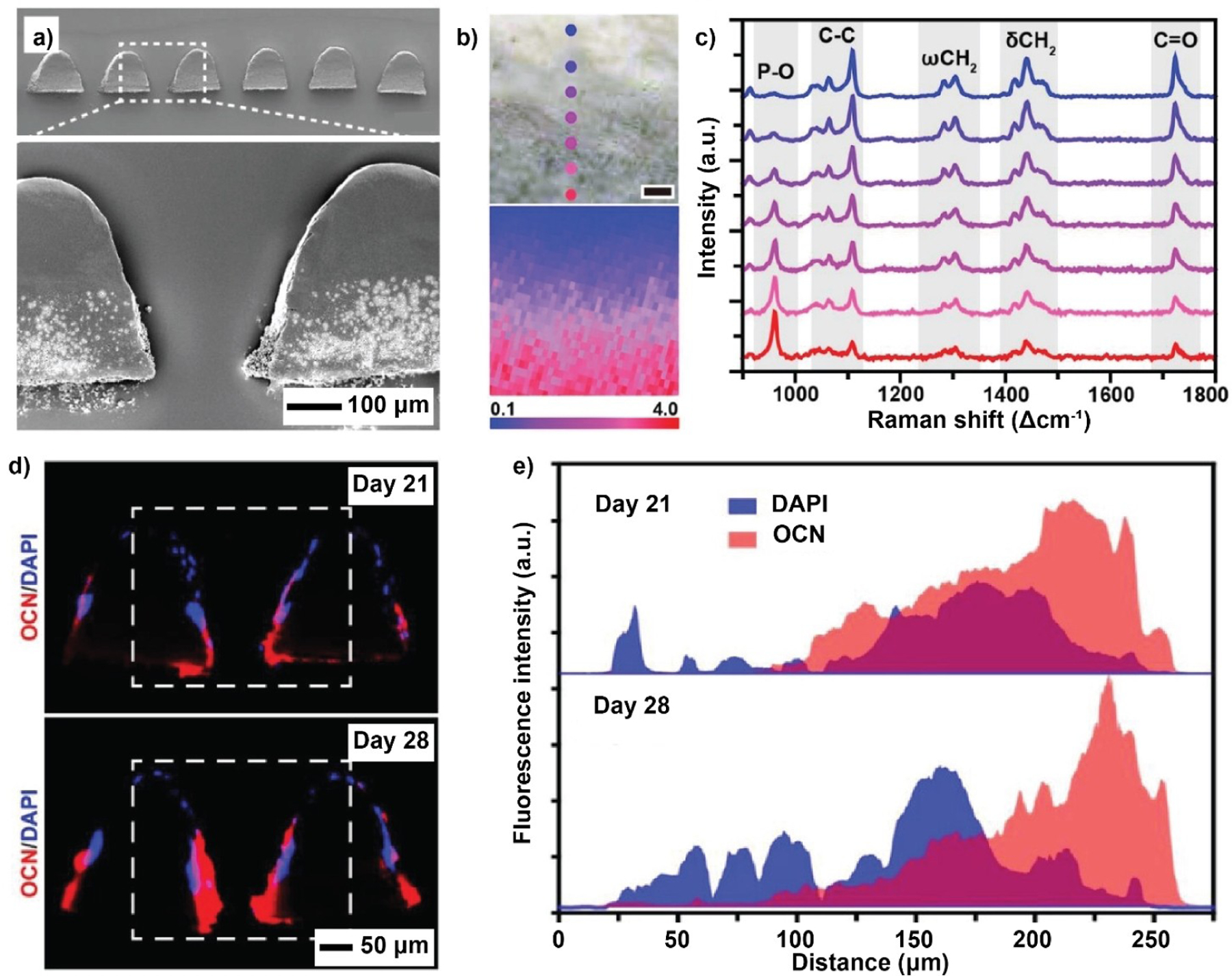
a) Cross-sectional SEM images of a HAp/PCL nanocomposite with graded HAp density. The bright spots correspond to the HAp nanoparticles. b) Raman spectroscopy mapping of HAp distribution in the graded region (scale bar: 10 μm). The color correlates to the intensity ratio between the characteristic peaks of HAp (P—O stretch at 960 cm^−1^) and PCL (C=O stretch at 1724 cm^−1^), respectively. c) Representative Raman spectra recorded from different sites indicated in (b). d) Cross-sectional fluorescence images of ASCs in the scaffold after 21 and 28 days of osteogenic differentiation. OCN and cell nuclei were stained to give red and blue fluorescence, respectively. e) Quantitative analysis of OCN expression along the channel, corresponding to the images in (d). Reproduced with permission from ref. [39]. Copyright 2021 Wiley-VCH.

The success in guiding the differentiation of stem cells inspired the rational development of other functionally-graded nanocomposites for recreating connective tissues with proper gradient compositions and optimized anatomical structures. To this end, various types of nanocomposites consisting of HAp nanoparticles dispersed in a biocompatible polymer matrix have been designed for tendon-to-bone repair.^[39,112,249]^ As the inorganic component of bones and teeth, HAp is widely used to regulate the osteogenic differentiation of stem cells due to its superior osteoinductivity. In one study, our group developed a HAp/PCL nanocomposite with a gradient in HAp particle density. In a typical process, a nanocomposite slab with a HAp-graded zone in the middle was fabricated by leveraging swelling-induced diffusion. An array of funnel-shaped microchannels was laser-drilled through the slab to allow for ASC infiltration, growth, and differentiation. As shown by the cross-sectional SEM image in Figure 18a, the density of HAp nanoparticles increased toward the bottom of the scaffold. The graded distribution of HAp was further confirmed by the gradual increase in the relative intensity of its characteristic P–O stretching mode, as depicted in the Raman microscopy mapping in Figure 18b,c. The ASCs seeded in the channels were driven toward different phenotypes by the spatial gradation in biochemical and mechanical stimuli associated with the varying amounts of HAp in the nanocomposite. Enhanced expression of osteocalcin (OCN)–a differentiation marker of osteogenesis–at the bottom of the channel in the fluorescence image (Figure 18d,e) indicated more prevalent osteogenic differentiation of ASCs in the region with a higher HAp density. The graded osteogenesis was supposed to be induced by a combination of graded osteoconductivity, osteoinductivity, and mechanical properties arising from the HAp gradient in the nanocomposite.

In addition to the aforementioned synthetic polymers like PLGA and PCL, a variety of natural polymers, including collagen and its derivatives,^[57–59]^ chitosan,^[69,70]^ silk fibrin,^[60]^ alginate,^[71,72]^ and agarose,^[73,74]^ have also been employed as the matrix materials for interfacial tissue repair. These polymers possess inherent biocompatibility, with some of them serving as vital constituents in the natural ECM of osteo-cartilage tissue, rendering them ideal choices for scaffolds.^[69]^ However, as is the case for PLGA or PCL, they typically exhibit elastic moduli orders of magnitude lower than those of the tissues near the bone end of the transitional zone.^[60]^ More importantly, the polymers alone cannot guide stem cell differentiation. To address these challenges, it is necessary to obtain functionally-graded nanocomposites with features that facilitate the regeneration of the tissues by incorporating inorganic nanoparticles. To date, these hybrid nanomaterials have been successfully applied to not only osteochondral tissue regeneration but also bone and DEJ. When other nanofillers, such as SPIO nanoparticles, are utilized, graded nanocomposite-based scaffolds facilitate cardiac and neural tissue restoration.^[242]^

### 3.3. MOFs

Owing to their extensive porosity, significant surface area, and customizable surface chemistry, MOFs have emerged as a versatile platform for biomedical applications.^[250–252]^ As an inherently hybrid system, the metal ions/clusters and the organic ligands can be judiciously selected to serve distinct theranostic roles. When used as nanoscale carriers, MOFs allow for the simultaneous delivery of various therapeutic agents, in addition to a high loading capacity and a robust method for reproducible surface modification.^[253]^ All these attributes play a critical role in minimizing off-target drug delivery and facilitating the accumulation of therapeutic agents at the intended site of action, which is especially important in enhancing the therapeutic window for cancer.^[254]^ Their ability to enhance the efficacy, specificity, and safety of therapeutic agents offers an attractive route to the development of advanced regimes.

**Figure 19.**
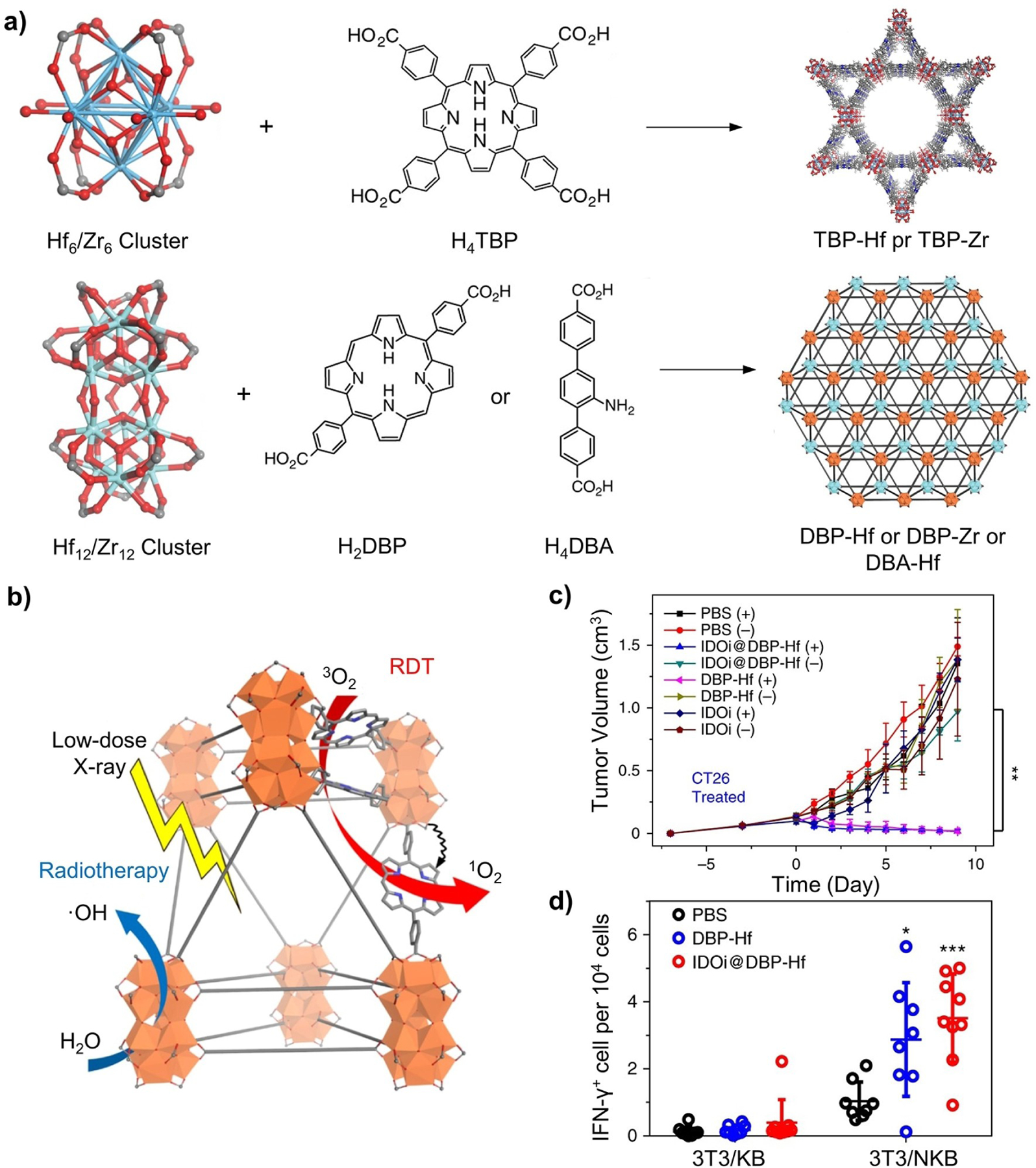
a) Schematics of the metal clusters, porphyrin-based ligands, and resultant Hf/Zr-based MOFs. b) Illustration of the therapeutic process involving X-ray RT-RDT enabled by the MOFs. c) Tumor growth profiles of mice with CT26 tumors following different treatment modalities. d) IFN-γ-producing T cells detected by enzyme-linked immunosorbent spot analysis. Reproduced with permission from ref. [40]. Copyright 2018 Springer Nature.

A recent study demonstrated the synthesis and utilization of a class of nanoscale MOFs to achieve low-dose X-ray RT-RDT, together with checkpoint blockade immunotherapy.^[40]^ The two representative MOFs, 5,15-di(*p*-benzoato)porphyrin-Hf/Zr (DBP-Hf/Zr) and 5,10,15,20-tetra(*p*-benzoato)porphyrin-Hf/Zr (TBP-Hf/Zr), were synthesized using Hf/Zr clusters and porphyrin-based ligands (Figure 19a). The Hf/Zr clusters can effectively capture X-ray photons to induce radiotherapy by producing ·OH radicals whereas the porphyrin units can serve as a photo-sensitizer for radiodynamic therapy by generating singlet oxygen (Figure 19b). At a minimal X-ray dose, these two MOFs could be used to efficiently eradicate various types of cancer cells both in vitro and in vivo. Enhanced immune infiltration and the presence of functional T and B cells indicated that RT-RDT also elicited an antitumor immune response (Figure 19c,d). The incorporation of a small molecule inhibitor for indoleamine 2,3-dioxygenase within the channels or pores of DBP-Hf/Zr, primarily through π–π interactions, further enabled checkpoint blockade immunotherapy to enhance the therapeutic outcomes in mouse models for breast and colorectal cancers. This combination approach resulted in an unprecedented 100 % abscopal response, as indicated by regression or rejection in both treated/irradiated and untreated/unirradiated tumors. This work confirms that the MOFs rationally constructed from the coordination of organic ligands and inorganic heavy metal ions and then loaded with small-molecule inhibitors hold great potential to improve the enduring response rate in cancer treatment.

A similar nanomaterial containing the porphyrin photo-sensitizer was also reported for PDT, where the resultant hypoxia condition was utilized to activate a bioreductive prodrug banoxantrone (AQ4N) and thus achieve combination therapy.^[49]^ Using strain-promoted azide-alkyne cycloaddition, the surface of the MOFs was further adorned with a dense PEG layer, enhancing their dispersion in the physiological milieu and boosting their therapeutic performance. Experiments conducted both in vitro and in vivo demonstrated that such MOFs could effectively exacerbate intracellular and tumor hypoxia. The escalation in hypoxia-activated the cytotoxic potential of AQ4N through a sequential cascade, leading to a synergistic therapeutic effect that combined PDT induction and hypoxia-activated treatment.

Other inorganic components can also be included in MOFs to further increase their figure of merit for cancer treatment. For example, up-conversion nanoparticles have been encapsulated in porphyrin-based MOFs to enable PDT with NIR irradiation.^[42]^ The core–shell nanoparticle was also loaded with an anticancer drug such as tirapazamine (TPZ) to amplify treatment effectiveness and yield a more robust therapeutic impact. The MOFs effectively integrate NIR-triggered PDT with hypoxia-induced chemotherapy, effectively treating hypoxic tumors through a synergistic effect. The combination therapy not only eradicated primary tumors but also suppressed distant tumors by harnessing systemic antitumor immunity facilitated by the chemo-PDT approach in BALB/c mice bearing CT26 tumors. Importantly, substantial increases in the infiltration of CD4 + T cells, CD8 +T cells, CD45 +cells, and NK cells were observed in both primary and distant tumors in the TPZ/UCSs+PD-L1 group after the PDT-mediated treatment.

In summary, nanostructured MOFs present distinct advantages in terms of biocompatibility and broad utility for diverse cancer therapies due to their compositional versatility, considerable porosity, and modifiable structure and function. Their straightforward synthesis, customization, and manageable manipulation of size and structure have contributed to their success in an array of biomedical applications, including cancer treatment.^[255]^ In addition, the use of organic molecules possessing inherent fluorescence properties allows for multifaceted imaging capabilities within a single framework. The resulting MOFs can exhibit impressive imaging sensitivity with high spatial resolution. Furthermore, careful selection of metal ions and organic ligands facilitates the direct creation of MOFs-based multifunctional systems, enabling the integration of imaging with therapy. Overall, MOFs stand as a strong candidate for further exploration in biomedical research. Given their relatively new status in the field of biomedicine, biocompatibility warrants attention before clinical implementation.^[256]^

### 3.4. Biohybrids

As an advantage, biohybrids inherit the bio-responsiveness typical of living organisms.^[136]^ This “smart” feature implies that they can detect biological signals and/or pathological abnormalities, such as pH, redox, and hypoxia conditions, as well as the presence of enzymes, ions, and signaling molecules in the microenvironment, and can invoke dynamic adaptation behavior to maintain homeostasis.^[257]^ In particular, many cells or organisms have demonstrated distinctive motility functions, inspiring researchers to engineer them as drug-delivery vehicles.^[258]^ From the perspective of material science, living cells or bacteria, such as *Lactococcus lactis*,^[259]^ could effectively assemble and grow into bone, bacterial biofilms, as well as other natural materials by integrating precursors into complex molecules, playing the role of dynamic smart material.^[146]^ As a major advantage over manmade materials, living organisms possess innate compatibility and stability in the complex biological environment. Certain types of living cells and bacteria exhibit complex cellular functions that are useful for therapeutic purposes. For instance, leukocytes are capable of transversing across inflamed endothelium through receptor-ligand interaction,^[260]^ potentially delivering drugs across this boundary. Numerous immunogenic antigens are present on bacteria membranes,^[261]^ making them appealing vaccination candidates. On the other hand, to match modern clinical needs, therapeutic agents are expected to demonstrate easily tunable physiochemical properties or other abiotic functionalities foreign to native cells. With their unique and tunable electric, magnetic, thermal, photonic, mechanical, and biochemical properties, inorganic materials should be integrated with living organisms to enhance their performance. Therefore, it is advantageous to incorporate inorganic nanostructures and living organisms into a hybrid system to harness the complete spectrum of properties afforded by both components.

Internalization of inorganic nanostructures is a crucial avenue to constructing cell-based biohybrids. Once internalized, the nanostructures interact with the cells by two major mechanisms. In the first scheme, the nanostructures directly augment the cell with its intrinsic properties, such as ferromagnetism,^[262]^ photoluminescence,^[263]^ and photothermal capability,^[264]^ enabling contrast enhancement, cell/virus tracking, and combination therapy. To this end, T cells were equipped with AuNPs for immunotherapy.^[23]^ The internalized AuNPs enabled dual-mode CT/fluorescence imaging of the subcutaneously injected T cells in vivo, allowing for live monitoring of the biodistribution of these cells and potentially elucidating their functional mechanisms.

**Figure 20.**
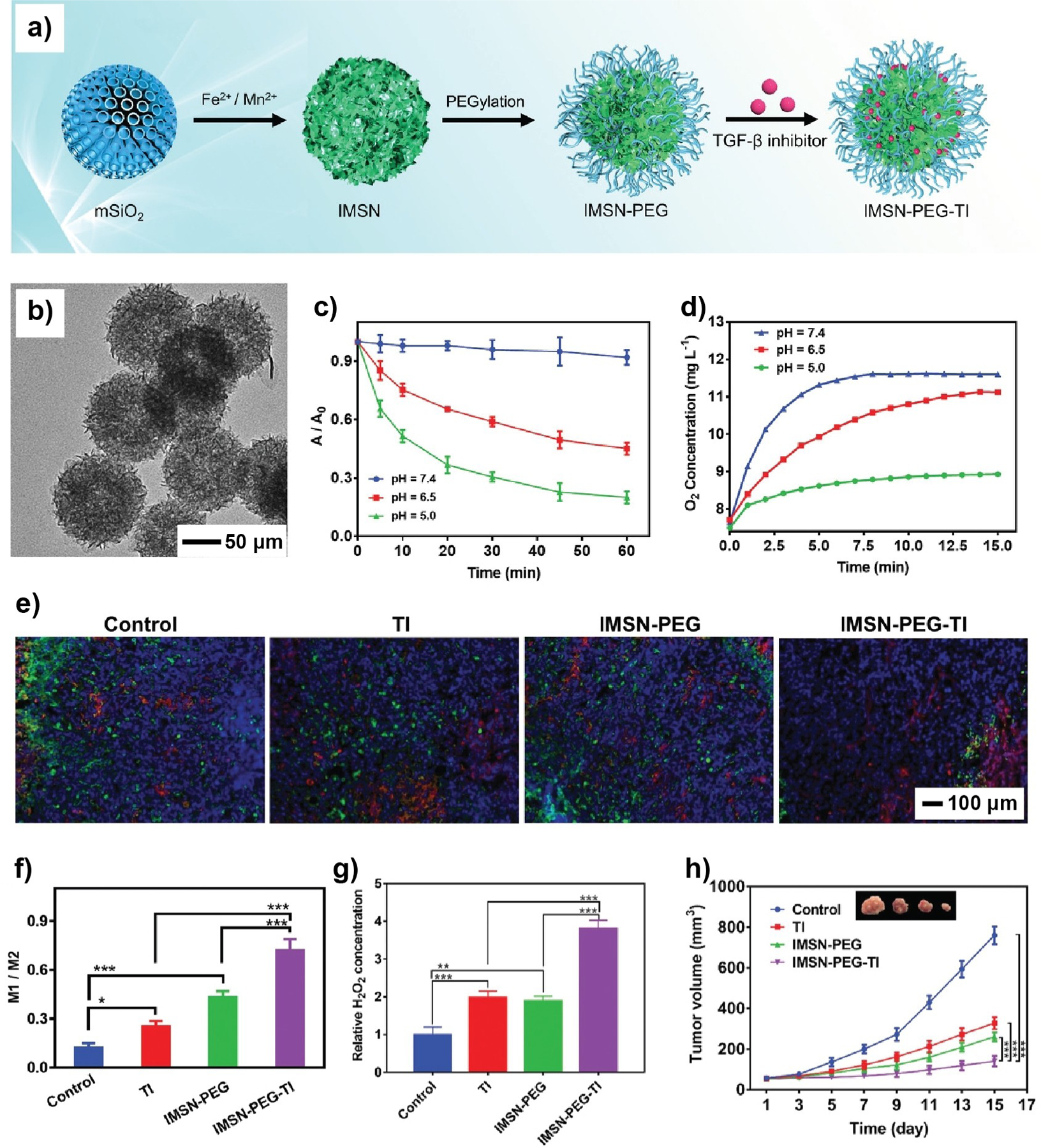
a) Schematic showing the synthesis of IMSN-PEG-TI for cancer therapy. b) TEM image of the nanoparticles. c) Normalized absorbance of MB (A/A_0_) and d) generation of O_2_ after adding IMSN (100 μg mL^−1^) and H_2_O_2_ (40 mM) in PBS buffer at pH of 5.0, 6.5, and 7.4. e) Immunofluorescence images of M1 and M2 macrophages in tumor tissues (red: M1 macrophages, green: M2 macrophages). f) The ratio of M1 to M2 macrophages. g) In vitro measurement of the generation of H_2_O_2_ when the IMSN-PEG-TI was injected into a tumor model. h) Tumor volume of CT26-tumor-bearing mice after different treatments for 15 days. Reproduced with permission from ref. [48]. Copyright 2020 Wiley-VCH.

On the other hand, internalization of inorganic nanostructures can activate associated signal pathways to regulate cell fate and biological processes, such as directing the differentiation of engineered stem cells for regenerative medicine and the activation of immune cells for cancer immunotherapy.^[137]^ One study reported the fabrication of a biohybrid system with catalytic nanoparticles and activated macrophages interacting synergistically for effective cancer treatment.^[48]^ PEGylated IMSN particles loaded with TGF-β inhibitor (TI) (collectively, denoted IMSN-PEG-TI) were constructed as a platform for catalytic therapy (Figure 20a,b). In the tumor microenvironment, these hybrid nanoparticles act as both peroxidase and catalase, decomposing H_2_O_2_ into ·OH radicals and O_2_, respectively. The catalytic capabilities of the nanoparticles were evaluated in vitro through a methylene blue degradation assay for peroxidase-like activity and dissolved oxygen generation assay for catalase-like activity, respectively. In a buffer mimicking the tumor acidic environment (pH= 6.5), the nanoparticles demonstrated both effective reduction of methylene blue by 54.9 % (Figure 20c) and adequate generation of O_2_ (Figure 20d). When employed as a therapeutic agent, their activity would quickly relieve tumor hypoxia and generate radicals that eventually cause apoptosis. However, the overall in vivo effectiveness of this treatment was limited by the rapid draining of endogenous H_2_O_2_, leading to compromised catalytic therapy performances. This is where the recruitment of macrophages became crucial to the system. Besides the catalytic activities, the nanoparticles could regulate the immune microenvironment and induce tumor-associated macrophages (TAMs) to polarize from M2-type into M1. Upon injection of the nanoparticles, a clear decrease in the M2 population (green color) and a simultaneous increase in the M1 population (red color) were observed in the immunofluorescence image of the tumor tissue (Figure 20e,f). The reprogrammed TAMs efficiently generated H_2_O_2_ (Figure 20g), supplementing the reactants for the catalytic reactions and thus augmenting the catalytic treatment. When applied to a mouse tumor model, this biohybrid system achieved a high tumor suppression rate of 87.5 %. Compared to the control groups, treatment with the biohybrid system resulted in the most effective tumor volume reduction (Figure 20h). The complementary functions of the inorganic nanostructures (IMSN-PEG-TIs) and the living component (macrophages) hold the key to the high performance.

A similar strategy was also used to fabricate biohybrids for immunotherapy. For example, certain species of *Escherichia coli* (*E. coli*) exhibit the capability to sense blue light and release the encoded flagellin B. Leveraging these properties, a bacteria-based biohybrid was constructed with *E. coli* and lanthanide UCNPs for optogenetic cancer immunotherapy.^[64]^ In addition, an “immune guide” based on magnetite nanoparticles was developed to recruit and activate macrophages for enhanced immunotherapies.^[62]^

Alternatively, the nanostructures can be enclosed by the membranes derived from cells to form a biohybrid system. This strategy usually involves the acquisition of cellular products to harness their native properties (e.g., biocompatibility, selectivity, sensitivity, and spatiotemporal control). It is particularly effective in camouflaging inorganic nanostructures from the immune response of the target organism toward exogenous materials.^[265]^ The proteins and polysaccharides on the cell membranes prevent these nanostructures from being attacked by the immune system, enhancing their biocompatibility, blood circulation, biodistribution, and duration of action.^[266]^ Erythrocyte membranes are most frequently employed for this purpose due to the simplicity of extraction,^[262,264,267]^ while membranes from other sources, such as leukocytes, platelets, cancer cells, exosomes, and bacteria, usually adopt a more active role in interacting with the physiological environment.^[268]^ Many of them have homing or targeting capability. For example, they can bind specifically to circulating cells capable of traversing to different tissues and crossing highly selective biological boundaries. One study reported the fabrication of a biohybrid with SPIO nanoparticles coated with platelet-derived vesicles (PLT-vesicles).^[31]^ The resultant system not only had an extended circulation time due to the inherent biocompatibility of PLT-vesicles but also inherited the cancer-targeting capability of platelets. As such, the biohybrid effectively accumulated at the tumor site, leading to enhanced MRI contrast and photothermal therapy. The membranes can also be collected to construct self-propelling biohybrid nanomotors.

Cell-in-shell design has also received increased attention in recent years. This hybridization scheme aims to construct an exoskeleton structure on the surface of a living cell to afford resistance to adverse environmental stimuli and augment its performance. Referred to as “SupraCells”, mammalian cells were encapsulated in inorganic nanomaterials such as MOFs, Fe_3_O_4_, and mesoporous SiO_2_.^[269]^ Although the SupraCells had enhanced resistance to mechanical stress and against endo- and exogenous stimuli, it was unclear whether the continuous presence of the exoskeletons would impede their interactions with ECM, especially for in vivo applications. A recent report described a method to enclose NSCs in hydrogen-bonded organic frameworks (HOFs) to improve their transplantation success rate.^[63]^ Along with the effectiveness in protecting the highly sensitive NSCs, the disintegration of the HOFs exoskeleton could be spatiotemporally controlled by irradiation with NIR light that dissociated the thermal-responsive hydrogen bonds, releasing the enclosed cells as commanded. This strategy might inspire future generations of cell-exoskeleton hybrid systems. Overall, cell-in-shell biohybrids remain an appealing concept that calls for further investigation on their behavior in in vivo environments and exploration of their biomedical applications.

## 4. Concluding Remarks

In response to the demands from diverse biomedical applications, spanning from non-invasive diagnosis to combination therapy and tissue regeneration, hybrid nanomaterials offer an effective, versatile, and customizable solution. The integration of inorganic and organic materials into a hybrid system not only preserves the distinct merits of each component but also produces additional features and/or synergistic effects to improve the outcome.^[6b]^ This review article offers a comprehensive analysis of the concept of material hybridization, including a discussion of the fabrication methods in the context of hybrid nanostructures, nanocomposites, MOFs, and biohybrids, together with high-lights of these hybrid nanomaterials in a spectrum of biomedical applications. Despite significant progress in recent years, this interdisciplinary field of research still faces several challenges, including *i*) identification and rational design of the inorganic and organic components for a hybrid system to best suit the biomedical application; *ii*) profound understanding of the interactions between a hybrid system and the biological world at different levels, including proteins, cells, tissues, organs, and whole body, to optimize the performance; and *iii*) development of effective, reproducible, and scalable methods for the production of hybrid nanomaterials.

The rational selection of proper inorganic and organic components represents the first and most important step in developing the hybrid system for a biomedical application.^[270]^ In general, the hybrid system should not impose any harmful impact on healthy tissues/organs, making biocompatibility the most important factor to consider when choosing the constituents. At the moment, however, this key issue is often ignored as most studies simply take whatever is available to achieve the properties, including acoustic, optical, electrical, magnetic, thermal, and/or mechanical, required for the proposed application. These properties certainly play a crucial role in defining the application. For instance, to achieve image-guided therapy, it is instrumental to focus on inorganic components with MRI capability and organic components with high loading efficiency and capacity for the construction of a hybrid system. In this regard, the primary determinant in identifying the proper inorganic and organic components is driven by the application. However, one must keep in mind that the ultimate merit of a biomaterial is determined by its biocompatibility or biosafety as long as it is intended for use in vivo.^[271]^

Once the inorganic and organic components have been specified, the next challenge is to systematically evaluate and optimize the performance by adjusting all the possible parameters involved, a process intricately related to the nano-bio interactions. In principle, the biological information progressively flows from proteins to cells and then tissues. As one of the fundamental biological factors balancing chemistry and biology, proteins serve as crucial mediators and participants in almost all nano-bio interactions.^[272]^ By leveraging the strategies of immobilization, conjugation, crosslinking, and/or self-assembly, proteins can be added to optimize the biological functions of a hybrid system. This process ensures the stability of the proteins while optimizing the biological functionality of the hybrid system. For instance, hybrid nanomaterials can be functionalized with cell-penetrating peptides to effectively pass through cell membranes in vitro and tissue barriers of in vivo models, enhancing the effectiveness in diagnosis and treatment. In addition to proteins, natural membranes can serve as a major contributor to the engineering of nano-bio interface. Besides, cells often serve as the primary subjects for in vitro exploration, and the cell-material interaction imposes a stronger demand to determine the specific parameters of a hybrid system. To this end, the distinct cell types may call for different sizes and shapes for the hybrid nanoparticles to achieve faster cellular internalization. This requirement, in turn, necessitates precise controls over the inorganic and organic components, as well as their hybridization specifics. In the end, all hybrid nanomaterials need to be evaluated and optimized in vivo for applications related to targeted delivery, immune evasion, circulation transport, and accumulation in tissues.

After optimizing all the unique parameters of a hybrid system for a specific application, the next challenge would be its reproducible and scalable production. While hybrid nanomaterials can now be prepared using laboratory proto-cols, their large-scale production for clinical applications is yet to be explored.^[273]^ To enable clinical translation of a hybrid nanomaterial, it is essential to shift from small-scale synthesis to large-scale production without compromising the properties. To achieve large-scale and cost-effective production at high efficiency, it is important to initiate or enhance collaboration among researchers from disciplines as diverse as chemistry, chemical engineering, materials science, biology, and medicine.^[274]^ It is the persistent improvement in the understanding, production, application, feedback, and refinement of hybrid nanomaterials that will pave the way for new prospects in human health.

## Figures and Tables

**Table 1:** Summary of inorganic components, together with their major properties, that have been exploited to construct hybrid nanomaterials for biomedical applications.

Inorganic components	Major properties	Biomedical applications	Refs.
Au nanostructures, including nanoparticles (AuNPs), nanorods (AuNRs), and nanocages (AuNCs)	Bio-inertness; strong and tunable optical absorption and scattering in the visible and near-infrared (NIR) regions; easy and robust bioconjugation with thiol- or disulfide-based ligands; and plenty of methods for colloidal synthesis	Nanoscale carriers for drug delivery; substrates for optical sensing; contrast agents for X-ray computed tomography (CT), surface-enhanced Raman scattering (SERS), and photoacoustic (PA) imaging; transducers for photothermal heating and localized hyperthermia; and theranostic agents for image-guided therapy	^[18–23]^
Ag nanostructures, including nanoparticles (AgNPs) and nanowires (AgNWs)	Antimicrobial activity; tunable optical absorption and scattering in visible region; strong SERS activity; and superior electrical and thermal conductivity	Wound management; contrast agents for SERS imaging; SERS tags for multiplex assay; and fabrication of flexible bioelectronics	^[24]^
Pd nanostructures such as nanocubes	Strong optical absorption for photothermal heating and catalytic activity	Localized heating for would management	^[25]^
Pt nanostructures such as nanoparticles	Catalytic activity	Fabrication of nanomotors	^[26]^
Quantum dots (QDs) such as those based on PbS	Size-dependent properties (e.g., emission wavelength) and photostability	Fluorescence imaging and optical tracking	^[27]^
Superparamagnetic nanoparticles such as those based on iron oxides	Magnetic activity and localized heating under an alternating magnetic field	Contrast agent for magnetic resonance imaging (MRI); separation and purification; magnetoresistance sensing; actuating; externally-guided motion or delivery; and localized hyperthermia	^[28–33]^
Mesoporous SiO_2_ nanoparticles (MSNPs)	Tunable pore size and high specific surface area	Controlled release and drug delivery	^[34,35]^
TiO_2_ nanorods	Optical responsiveness and catalytic activity	Light-powered nanomotors	^[36]^
MnO_2_ nanoparticles	Catalytic activity	Biodegradable nanomotors	^[37]^
CaO_2_ nanoparticles	Generation of O_2_	Anti-inflammation during wound healing	^[38]^
Hydroxyapatite (HAp) nanoparticles	High mechanical strength, biocompatibility, and osteoconductivity	Nanocomposites and graded scaffolds for tissue repair or regeneration	^[39]^
Hf/Zr clusters	Capture of X-ray photons	Low-dose X-ray radiotherapy and radiodynamic therapy	^[40]^
Carbon nanotubes (CNTs)	High mechanical strength, electrical conductivity, and specific surface area; NIR fluorescence	Fillers for nanocomposites; carriers for drug delivery; and contrast agents for optical and PA imaging	^[41]^
Up-conversion nanoparticles (UCNPs)	Emission of light at shorter wavelengths than incident lights without blinking	Photodynamic therapy and optical tracking	^[42]^
Gd-doped Au nanoparticles	Magnetic activity and optical responsiveness	Contrast agents for multi-modal imaging and image-guided therapy	^[43]^
Gd-doped CuS nanoparticles	Magnetic activity and optical absorption	Contrast agents for multi-modal imaging; localized hyperthermia; and image-guided therapy	^[44]^
Cu nanoparticles	Radioactivity for ^64^Cu	Probe for positron emission tomography (PET)	^[45,46]^
Graphene and graphene oxide (GO)	Flexibility and structural reinforcement; catalytic activity; and conductivity	Drug delivery and tissue repair or regeneration	^[47]^
Iron manganese silicate nanoparticles (IMSN)	Catalytic activity	Catalytic therapy and immunotherapy	^[48]^

**Table 2:** Summary of organic components, together with their major properties, that have been exploited to construct hybrid nanomaterials for biomedical applications.

Organic components	Major properties	Biomedical applications	Refs.
Small molecules or ions such as those based on thiols, disulfides, silanes, citrate, and halide ions	Diverse terminal groups in terms of charge, hydro-phobicity/philicity, and reactivity; and ability to form self-assembled monolayers (SAMs)	Colloidal stabilizers; surface capping agents; interfacial engineering; surface coupling; and conjugation of targeting ligands	^[18,19,42,49]^
Biocompatible and biodegradable polymers such as those based on polyesters, including poly(lactic-*co*-glycolic acid) (PLGA) and polycaprolactone (PCL)	Approved by FDA for human use; controllable degradation kinetics; suitable for casting films and electrospinning of nanofibers	Controlled release; carriers for drug delivery; wound management; matrices for nanocomposites; and graded scaffolds	^[39,47,50–54]^
Biomacromolecules such as proteins, enzymes, and antibodies	Biological activities and targeting capability	Targeted delivery and surface modification for sensing and multiplex assay	^[18,32,53,55–60]^
Poly(ethylene glycol) (PEG)	Highly stable and heavily solvated by water	PEGylation; resistance to protein adsorption; and holding of water	^[18,25,37,49]^
Phase-change materials (PCMs) such as those based on fatty alcohols or fatty acids	Tunable sharp melting points; involvement of heat exchange during phase transition; and change in diffusivity	Carriers for drug delivery and gating materials for controlled release	^[61]^
Living organism-derived materials such as cell and bacteria membranes; cell excreted liposomes; and exosomes	Innate biocompatibility and bioactivity; and targeting capabilities	Carriers for drug delivery; cancer immunotherapy; and regenerative medicine	^[31,48,62–64]^
Photosensitizers such as porphyrin, HPPH, UV initiators (2,2’-azobis[2-(2-imidazolin-2-yl) propane] dihydrochloride (AIPH)	Decompose rapidly under thermal or irradiation stimulation to generate alkyl radicals or reactive oxygen species (ROS)	Diagnostic imaging; controlled release for the treatment of hypoxic cancer; and antimicrobial photodynamic therapy	^[40,49,61]^
Photothermal polymers such as polydopamine (PDA)	Photothermal conversion; broad absorption spectrum including NIR light; biocompatibility; and biodegradability	Cancer photothermal therapy and carriers for drug delivery	^[65]^
Stimuli-responsive polymers such as thermal-responsive polymers, including poly(N-isopropylacrylamide) (pNIPAAm) and its derivatives, as well as Pluronics	Smart thermo-responsiveness and good biocompatibility	Molecular encapsulation for drug delivery and controlled release; smart actuators for living organism delivery; and wound management for tissue engineering	^[19,21–22,33,66]^
Zwitterionic molecules	Resist non-specific protein adsorption; minimize nonspecific protein adsorption and nonspecific cell uptake; and good biocompatibility	Tissue engineering scaffolds; resistance to protein adsorption; holding of water; and wound management	^[67]^
Thermoplastic polymers such as cyclic olefin copolymer (COC), polycarbonate (PC), and polystyrene (PS)	High mechanical degradation resistance; high strength and rigidity; and high transparency	Implantable fiber probe; soft robotics for cell delivery; and matrices for nanocomposites	^[24,33,68]^
Silicone polymers such as polydimethylsiloxane (PDMS)	High flexibility; good biocompatibility; and high mechanical degradation-resistance	Stretchable bioelectronics for neuroprosthetic applications; matrices for nanocomposites; tissue engineering scaffolds; and implantable devices	^[24,26]^
Polysaccharides such as agarose, chitosan, and hyaluronic acid	Good biocompatibility and biodegradability; hydrophilicity; and ability to stabilize nanoparticles	Controlled release; carriers for drug delivery; wound management; and nanocomposite matrices	^[69–75]^
Molecular imaging probes such as Raman active molecules (*trans-*1,2-bis(4-pyridyl)-ethylene) and aggregation-induced emission luminogens (AIEgens)	Unique imaging properties, such as Raman signature; enhancement in fluorescence intensity from a dispersed (solubilized) state to an aggregated state; and good photostability	Molecular imaging for monitoring; cell labeling and tracking; and photodynamic therapy	^[43,76]^

## Data Availability

The data that support the findings of this study are available from the corresponding author upon reasonable request.
